# Risk factors, resistance profiles, and clinical outcomes of *Klebsiella pneumoniae* infection in cancer patients: a retrospective study

**DOI:** 10.3389/fcimb.2026.1908965

**Published:** 2026-08-26

**Authors:** Shulei Zhang, Jingwen Wang, Shuo Zhang, Shuping Lu, Miaosu Chang, Wei Xu, Qingmei Zhang, Mei Zhu

**Affiliations:** 1 Department of Clinical Laboratory, The Fourth Affiliated Hospital of Anhui Medical University, ChaoHu, Anhui, China; 2 Department of Blood Transfusion, The First Affiliated Hospital of Anhui Medical University, Hefei, Anhui, China; 3 Department of Anesthesiology, The Fourth Affiliated Hospital of Anhui Medical University, ChaoHu, Anhui, China

**Keywords:** cancer patients, *Klebsiella pneumoniae*, multidrug resistance, prediction model, risk factors

## Abstract

**Background:**

Cancer patients are highly susceptible to *Klebsiella pneumoniae* (KP) infection, yet integrated analyses considering infection-site resistance patterns, cancer-type heterogeneity, and host risk factors remain limited.

**Methods:**

We retrospectively analyzed 300 adult cancer patients treated between October 2023 and November 2024, including 153 with KP infection and 147 uninfected controls. Data on demographics, comorbidities, invasive procedures, laboratory findings, antimicrobial resistance, treatment patterns, and outcomes were collected. Independent risk factors were identified using multivariable logistic regression, and a predictive nomogram was developed and validated.

**Results:**

The respiratory tract was the most common infection site (60.8%), followed by urinary tract (14.4%) and bloodstream (10.4%). Urinary isolates exhibited the highest rates of multidrug-resistant KP (MDR-KP, 69.6%) and extended-spectrum β-lactamase-producing KP (ESBL-KP, 60.9%). Multivariate analysis identified hypoproteinemia (odds ratio [OR] = 2.233, 95% confidence interval [CI] 1.222-4.077, *P =* 0.009), percutaneous catheter drainage (OR = 1.995, 95% CI 1.050-3.790, *P* = 0.035), and radiotherapy (OR = 3.556, 95% CI 1.507-8.392, *P* = 0.004) as independent factors associated with KP infection. Recent hospitalization was independently associated with both MDR-KP (OR = 3.257, 95% CI 1.227-8.649, *P* = 0.018) and ESBL-KP (OR = 3.101, 95% CI 1.151-8.355, *P* = 0.025). A nomogram incorporating these predictors demonstrated strong discriminative ability (area under the curve = 0.702) and clinical utility.

**Conclusion:**

Statistical models indicate that KP infection in cancer patients is likely linked to host immunity, invasive interventions, and treatment exposures; the nomogram can assist in early risk stratification and facilitate tailored stewardship.

## 1 Introduction

*Klebsiella pneumoniae* (KP) is a formidable opportunistic pathogen, frequently causing hospital- and community-acquired pneumonia, urinary tract infection, and bloodstream infection (BSI) (Ibrahim, 2023; Choby et al., 2020). It accounts for approximately 10% of all hospital-acquired infections, which have a global prevalence of 8.7%, underscoring its significant burden on healthcare systems (Mohd Asri et al., 2021). Compounding this threat is the rapid emergence and dissemination of multidrug-resistant (MDR) and extended-spectrum β-lactamase-producing (ESBL) strains. These resistant pathogens severely limit therapeutic options, complicate clinical management, and are associated with poorer patient outcomes (Asokan et al., 2025; Xu et al., 2025; El-Sherbiny et al., 2024b).

The risk posed by KP is not uniform across all patient groups, with cancer patients representing a particularly vulnerable population. Their susceptibility stems from a confluence of factors: immunosuppression caused by the underlying malignancy, myelosuppressive effects of chemotherapy and radiotherapy, and frequent breaches of anatomical barriers due to invasive procedures during prolonged hospitalization (Wang et al., 2025a; Rolston, 2017). Consequently, bacterial infections, including BSI, are major complications; BSI alone accounts for 20-30% of febrile episodes in neutropenic patients (Amanati et al., 2021). This impaired immunity renders cancer patients highly susceptible to KP and other nosocomial pathogens (Chetcuti Zammit et al., 2014; Shelke et al., 2024). Recent studies have characterized antimicrobial susceptibility and virulence among bacterial isolates from patients with cancer (El-Sherbiny et al., 2024b), while related work has evaluated plant-derived or nanoemulsion-based activity against MDR or extensively drug-resistant (XDR) organisms and cancer cell lines (Abdel Fattah et al., 2026; Ramadan et al., 2026; El-Sherbiny et al., 2024a). These studies advance microbiological and therapeutic understanding but do not address how routine clinical exposures, infection site, tumor type, resistance phenotype, treatment patterns, and outcomes intersect in adults with solid tumors (Allel et al., 2023; Nanayakkara et al., 2021).

To address these gaps, this retrospective study was designed to conduct a detailed clinical and microbiological characterization of KP infections in adult cancer patients. The study aimed to (1) describe the site-specific and cancer-type-specific resistance profiles; (2) identify factors associated with KP infection, MDR-KP, and ESBL-KP; (3) examine treatment patterns and clinical outcomes; and (4) develop an internally validated nomogram based on routinely available clinical variables. This integrated design was intended to support practical risk stratification and more informed empirical antimicrobial management in oncology care.

## 2 Materials and methods

### 2.1 Studying design

This retrospective study included 300 adults (≥18 years) with histologically or cytologically confirmed malignant tumors who were hospitalized at the First Affiliated Hospital of Anhui Medical University between October 2023 and November 2024. Inclusion criteria were: (1) age ≥18 years; (2) confirmed malignancy; (3) complete clinical and microbiological data; (4) culture-confirmed KP infection with compatible clinical findings for infection group; and (5) no clinical or microbiological evidence of infection for the control group. Exclusion criteria included: (1) colonization without clinical infection or polymicrobial infection; (2) incomplete data; (3) repeated hospitalizations (only the first admission was included); (4) hospital stay < 24 h; and (5) hematologic malignancies. Patients with hematologic malignancies were excluded because marrow disease and its treatment directly alter leukocyte-based inflammatory indices; restricting the cohort to solid tumors reduced etiologic heterogeneity in analyses that included these markers.

Eligible uninfected patients hospitalized during the same period formed the candidate control pool. The matched cohort (147 pairs; n = 294) was used to identify factors associated with KP infection, whereas all 153 infected patients were included in descriptive analyses of infection sites and resistance phenotypes. The study was conducted in accordance with the Declaration of Helsinki and approved by the Ethics Committee of the First Affiliated Hospital of Anhui Medical University.

### 2.2 Data collection

Clinical data were extracted from the hospital electronic medical record system. Variables included patient demographics (age, sex, and body mass index [BMI]), cancer diagnoses, metastasis, comorbidities and hypoproteinemia. Disease burden, severity, and functional status were assessed using the age-adjusted Charlson Comorbidity Index (aCCI), quick Sequential Organ Failure Assessment (qSOFA) score, and Activities of Daily Living (ADL) score, respectively. Data on invasive procedures, cancer-related treatments, concomitant medications, infection sources, antimicrobial susceptibility profiles, laboratory findings, intensive care unit (ICU) admission, length of stay (LOS), and clinical outcomes were also collected.

### 2.3 Variable definitions

The diagnostic criteria for KP infection were defined as follows: (1) a positive KP culture from a sterile site with compatible clinical symptoms; (2) In accordance with the Chinese Diagnostic Criteria for Hospital-Acquired Pneumonia and Ventilator-Associated Pneumonia in Adults (2018 Edition) and the Chinese Guidelines for the Diagnosis and Treatment of Community-Acquired Pneumonia in Adults (2018 Practical Edition), respiratory infection required a positive respiratory culture, new or worsening clinical features (e.g., fever, purulent sputum, or dyspnea), new or progressive radiographic infiltrates, and a documented clinical diagnosis of pneumonia requiring the initiation or escalation of KP-targeted antimicrobial therapy; (3) urinary tract infection diagnosed based on the Chinese Expert Consensus on the Diagnosis and Treatment of Urinary Tract Infections (2015 Edition) (Wang et al., 2025c); (4) BSI, defined as at least one positive blood culture accompanied by clinical signs and symptoms of bacteremia (Freifeld et al., 2011). KP isolates that did not fulfill these criteria were regarded as colonization (Howard-Anderson et al., 2022).

Community-acquired infection (CAI) was defined by a first positive culture obtained < 48 hours after admission, and hospital-acquired infection (HAI) was by a first positive culture obtained ≥ 48 after admission without evidence of infection on admission. Furthermore, infections directly linked to medical interventions—such as urinary, arterial, or venous catheters, blood transfusions, or surgical procedures—were classified as healthcare-associated infections (HCAI), irrespective of the 48-hour threshold (Haque et al., 2020; Li et al., 2023b). MDR-KP was defined as acquired resistance to at least one agent in three or more antimicrobial categories (Song et al., 2025; Ramadan et al., 2026). ESBL-KP was identified when the minimum inhibitory concentration (MIC) of ceftazidime, ceftriaxone, cefotaxime, or aztreonam was ≥ 2 mg/L (Wang et al., 2023a). XDR isolates were defined as non-susceptible to at least one agent in all but ≤ 2 antimicrobial categories; and pandrug-resistant (PDR) isolates were defined as non-susceptible to all agents in all antimicrobial categories (Abdel Fattah et al., 2026). Empirical antibiotic therapy (EAT) referred to antibiotics administered after specimen collection but before the availability of susceptibility results (Cheng et al., 2023). Combination antibiotic therapy was defined as the concurrent administration of two or more antimicrobial agents for more than 24 hours. Post-infection hospitalization duration was calculated as the period between the date of the first positive specimen collection and the date of discharge. Hypoalbuminemia was defined as a serum albumin level < 35 g/L at admission (Wang et al., 2023b).

### 2.4 Microbiological analysis

All KP isolates were processed in accordance with the National Clinical Laboratory Procedures of China (4th edition). Clinical samples were cultured on Columbia blood agar, Hemophilus-enriched chocolate agar, and MacConkey agar plates and incubated at 35 °C in 5% CO_2_ environment for 24–36 hours. Bacterial identification was performed using an automated matrix-assisted laser desorption/ionization time-of-flight mass spectrometry system (VITEK^®^ MS, bioMérieux, France). Antimicrobial susceptibility testing was conducted using AST-N13 or AST-N335 cards on the VITEK 2 Compact analyzer, and results were interpreted according to the Clinical and Laboratory Standards Institute (CLSI) M100, 2023 edition. *Escherichia coli* ATCC 25922 and *Pseudomonas aeruginosa* ATCC 27853 were included for quality control.

### 2.5 Statistical analysis

Statistical analyses were performed using IBM SPSS Statistics (version 27.0; IBM Corp., Armonk, NY, USA) and R software (version 4.5.1; R Foundation for Statistical Computing, Vienna, Austria). Categorical variables were presented as frequencies and percentages, and continuous variables as mean ± standard deviation (SD) or median (interquartile range [IQR]), as appropriate. Normality was assessed using the Shapiro-Wilk test. Group comparisons were performed using the independent-samples t-test or Mann-Whitney *U* test for continuous variables, and the chi-square test or Fisher’s exact test for categorical variables. Pairwise comparisons across cancer types or infection sites were adjusted using the Bonferroni correction.

A 1:1 propensity score matching (PSM) model used nearest-neighbor matching without replacement and included sex, age, BMI, qSOFA score at admission, cancer type, and metastatic status. A prespecified caliper of 0.03 on the propensity-score scale was selected to restrict poor matches while retaining 147 pairs (Bao et al., 2026). Covariate balance was assessed using standardized mean differences (SMDs), with SMD < 0.20 considers acceptable (Austin, 2009, 2011). Following PSM, variables with *P* < 0.20 in the univariate analysis were included in the multicollinearity assessment. Variables with a variance inflation factor > 5 were excluded. Multivariable logistic regression models were then constructed based on the matched cohort to identify independent risk factors for KP infection. Results are reported as odds ratios (ORs) with 95% confidence intervals (CIs).

A predictive nomogram was developed from the matched cohort and internally validated using 1,000 bootstrap resamples (the “rms” package). Model performance was comprehensively evaluated by discrimination (area under the receiver operating characteristic curve [AUC], the “pROC” package), calibration curves, and decision curve analysis (DCA, the “rmda” package). All statistical tests were two-sided, and *P* < 0.05 was considered statistically significant.

No data were missing for demographic, clinical, laboratory, or prediction-model variables. Antimicrobial susceptibility analyses used the available test result for each isolate because not every agent was routinely tested; no values were imputed. Agent-specific denominators are reported in **Supplementary Table 1**.

## 3 Results

### 3.1 Study cohort and baseline characteristics of KP infection.

**Figure 1:**
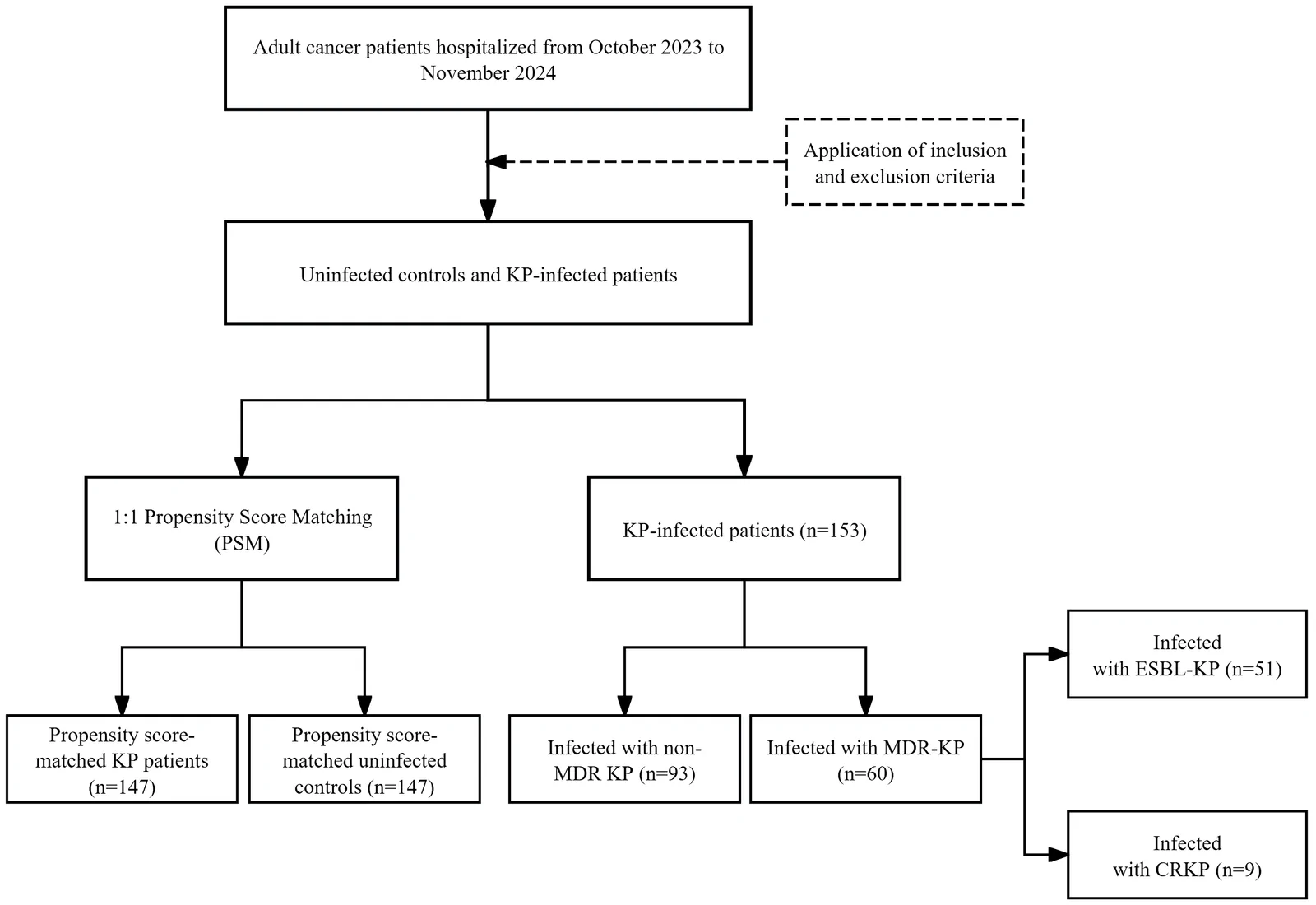
Flow diagram of patient selection and cohort construction.

This retrospective study enrolled 300 adult cancer patients, including 153 patients (51.0%) with a confirmed KP infection and 147 uninfected controls (49.0%). The KP-infected group included 60 patients (20.0%) with MDR-KP, 51 (17.0%) with ESBL-KP, and 9 (3.0%) with CRKP (**Figure 1**).

After matching, all included covariates had SMDs < 0.2, indicating the acceptable balance (**Supplementary Table 2**). Compared with uninfected controls (**Table 1**), KP-infected patients exhibited a greater burden of comorbidities, particularly Diabetes mellitus, digestive diseases, and hypoalbuminemia (all *P* < 0.05). Functional status was also poorer, as reflected by lower ADL scores at admission and discharge (all *P* ≤ 0.001). Invasive procedures, including urinary catheterization, percutaneous catheter drainage, ventilator use, tracheal cannulation, endoscopy, and recent surgery, were more frequently performed in the infected group (all *P* < 0.02). Laboratory findings indicated a markedly heightened inflammatory response in KP-infected patients, with significantly elevated systemic immune-inflammation index (SII), neutrophil-to-lymphocyte ratio (NLR), platelet-to-lymphocyte ratio (PLR), and monocyte-to-lymphocyte ratio (MLR), accompanied by increased white blood cell (WBC) and neutrophil counts (Neu) and decreased lymphocytes (Lym), hemoglobin (Hb), total protein (TP), and albumin (ALB) (all *P* ≤ 0.003). Mild increases in liver function markers were observed, whereas renal function did not differ significantly between groups. In addition, KP infection was associated with substantially higher hospitalization costs, medical expenses, and antibiotic expenditures (all *P* < 0.001).

**Table 1:** Clinical characteristics of the KP-infected and uninfected groups after propensity score matching.

Variables	Total (n=294)	KP only (n=147)	Uninfected(n=147)	*P*-value*
**Sex, M, No. (%)**	210 (71.4%)	101 (68.7%)	109 (74.1%)	0.302
**Age, y, median (IQR)**	66 (57, 73)	68 (59, 73)	66 (57, 71)	0.259
**BMI, median (IQR)**	21.26 (18.79, 23.88)	20.96 (18.04, 24.22)	21.36 (19.27, 23.66)	0.598
History, No. (%)				
Drinking	70 (23.8%)	37 (25.2%)	33 (22.4%)	0.584
Smoking	83 (28.2%)	46 (31.3%)	37 (25.2)	0.244
Hospitalization	205 (69.7%)	101 (68.7%)	104 (70.7%)	0.703
Cancer type, No. (%)				
Lung cancer	62 (21.1%)	32 (21.8%)	30 (20.4%)	0.775
Gastrointestinal cancer	95 (32.3%)	45 (30.6%)	50 (34.0%)	0.533
Hepatobiliary and Pancreatic cancer	59 (20.1%)	29 (19.7%)	30 (20.4%)	0.884
Others	76 (25.9%)	41 (27.9%)	35 (23.8%)	0.424
With metastasis	164 (55.8%)	82 (55.8%)	82 (55.8%)	1.000
Comorbid conditions, No. (%)				
Hypertension	106 (36.1%)	56 (38.1%)	50 (34.0%)	0.466
Diabetes mellitus	45 (15.3%)	30 (20.4%)	15 (10.2%)	**0.015**
Nervous system diseases	54 (18.4%)	32 (21.8%)	22 (15.0%)	0.132
Respiratory diseases	66 (22.4%)	33 (22.4%)	33 (22.4%)	1.000
Cardiovascular diseases	50 (17.0%)	29 (19.7%)	21 (14.3%)	0.214
Digestive diseases	28 (9.5%)	19 (12.9%)	9 (6.1%)	**0.047**
Chronic kidney diseases	46 (15.6%)	20 (13.6%)	26 (17.7%)	0.335
Chronic hepatic diseases	72 (24.5%)	34 (23.1%)	38 (36.0%)	0.587
**Hypoproteinemia, No. (%)**	122 (41.5%)	82 (55.8%)	40 (27.2%)	**<0.001**
**aCCI, median (IQR)**	8 (6, 9)	8 (6, 9)	8 (5. 9)	0.576
**ADL score at admission, median (IQR)**	95 (90, 100)	95 (80, 100)	100 (90, 100)	**0.001**
**ADL score at discharge, median (IQR)**	95 (85, 100)	90 (70, 100)	100 (90, 100)	**<0.001**
**qSOFA at admission ≥ 1, No. (%)**	77 (26.2%)	39 (38.5%)	38 (38.5%)	0.894
Invasive procedure, No. (%)				
Urinary catheter	44 (15.0%)	33 (22.4%)	11 (7.5%)	**<0.001**
Percutaneous catheter drainage	81 (27.6%)	56 (38.1%)	25 (17.0%)	**<0.001**
Venous and arterial catheter	94 (32.0%)	51 (34.7%)	43 (29.3%)	0.317
Use of ventilators	25 (8.5%)	20 (13.6%)	5 (3.4%)	**0.002**
Tracheal cannula	25 (8.5%)	20 (13.6%)	5 (3.4%)	**0.002**
Endoscope	43 (14.6%)	29 (19.7%)	14 (9.5%)	**0.013**
Others	16 (5.4%)	9 (6.1%)	7 (4.8%)	0.607
Surgery within 90 d	73 (24.8%)	48 (32.7%)	25 (17.0%)	**0.002**
Laboratory findings following infection, median (IQR)				
WBC, ×10^9/L	5.55 (4.49, 7.50)	6.50 (4.34, 9.89)	5.24 (4.50, 6.23)	**<0.001**
Neu, ×10^9/L	3.63 (2.76, 5.84)	4.76 (2.95, 8.24)	3.24 (2.75, 4.07)	**<0.001**
Lym, ×10^9/L	1.15 (0.67, 1.51)	0.78 (0.44, 1.37)	1.36 (1.03, 1.68)	**<0.001**
Mon, ×10^9/L	0.37 (0.26, 0.51)	0.39 (0.22, 0.57)	0.36 (0.28, 0.46)	0.617
PLT, ×10^9/L	159 (123, 224)	149 (111, 222)	166 (130, 231)	0.062
Hb, g/L	111 (96, 125)	105 (91, 121)	118 (101, 131)	**<0.001**
SII	554.20 (313.37, 1311.78)	960.54 (446.85, 1873.46)	401.74 (262.24, 705.98)	**<0.001**
NLR	3.37 (2.08, 8.07)	6.50 (2.92, 11.21)	2.38 (1.73, 3.57)	**<0.001**
PLR	157.83 (105.27, 246.03)	203.17 (121.88, 318.19)	131.88 (96.55, 180.82)	**<0.001**
MLR	0.33 (0.22, 0.57)	0.48 (0.27, 0.79)	0.26 (0.20, 0.36)	**<0.001**
ALT, U/L	18.0 (11.2, 28.0)	18.6 (12.0, 30.5)	16.8 (10.9, 25.1)	0.055
AST, U/L	23.9 (18.0, 37.0)	26.8 (18.0, 41.8)	22.1 (18.0, 30.9)	**0.032**
TB, μmol/L	13.31 (9.80, 19.55)	14.14 (9.75, 20.62)	12.68 (9.79, 17.86)	0.077
TP, g/L	62.3 (57.1, 67.5)	60.1 (55.5, 65.7)	63.3 (59.3, 68.8)	**0.003**
ALB, g/L	36.9 (33.2, 41.1)	34.9 (31.3, 38.6)	39.4 (35.5, 42.3)	**<0.001**
Cr, μmol/L	63.5 (54.0, 77.1)	62.5 (51.1, 80.4)	65.9 (56.0, 75.9)	0.271
Bun, mmol/L	5.29 (4.25, 7.08)	5.18 (4.22, 7.73)	5.37 (4.25, 6.68)	0.514
Related cost(dollars)^#^, median (IQR)				
Total cost	1772.7 (1070.3, 3357.2)	2772.0 (1564.2, 6724.2)	1315.3 (843.2, 2028.8)	**<0.001**
Medical fee	868.9 (391.0, 1527.8)	1216.8 (566.8, 2032.7)	645.7 (310.9, 104.9)	**<0.001**
Antibacterial agent cost	3.5 (0, 74.1)	45.2 (7.9, 216.6)	0 (0, 0)	**<0.001**
Tumor treatment, No. (%)				
Radiotherapy	35 (11.9%)	25 (17.0%)	10 (6.8%)	**0.007**
Chemotherapy	196 (66.7%)	88 (59.9%)	108 (73.5%)	**0.013**
Immunotherapy	104 (35.4%)	47 (32.0%)	57 (38.8%)	0.223
Targeted drug	144 (49.0%)	69 (46.9%)	75 (51.0%)	0.484
Herbal therapy	73 (24.8%)	37 (25.2%)	36 (24.5%)	0.893
Concomitant drugs, No. (%)				
Immunosuppressant	202 (68.7%)	116 (78.9%)	86 (58.5%)	**<0.001**
Hormone	199 (67.7%)	125 (85.0%)	74 (50.3%)	**<0.001**
Clinical outcomes				
LOS in ICU, median (IQR)	0 (0, 0)	0 (0, 0)	0 (0, 0)	**0.001**
ICU, No. (%)	14 (4.8%)	13 (8.8%)	1 (0.7%)	**0.001**
LOS, median (IQR)	8 (5, 14)	12 (8, 23)	5 (3, 8)	**<0.001**
Clinical stability, No. (%)	244 (83.0%)	119 (81.0%)	125 (85.0%)	0.352
On the mend, No. (%)	17 (5.8%)	9 (6.1%)	8 (5.4%)	0.803
Deterioration, No. (%)	16 (5.4%)	12 (8.2%)	4 (2.7%)	**0.040**
Automatic discharge or transfer, No. (%)	35 (11.9%)	23 (15.6%)	12 (8.2%)	**0.048**
Readmission, No. (%)	201 (68.4%)	93 (63.3%)	108 (73.5%)	0.060
Paliative care, No. (%)	26 (8.8%)	10 (6.8%)	16 (10.9%)	0.218

*P values are obtained using chi-square test or fisher’s exact test for categorical variables and the independent-samples t-test or Mann-Whitney *U* for continuous variables. ^#^1 dollar = 7.1138 Chinese Yuan as of September, 2025. Data are represented as No. (%), IQR and mean ± SD. Bold values indicate statistical significance (*P* < 0.05).

Surgery within 90 d, Surgery within 90 days; Mon, monocytes; Cr, creatinine; Bun, blood urea nitrogen.

### 3.2 Risk factors and clinical impact of antimicrobial resistance

Within the KP-infected cohort, baseline characteristics—including demographics, cancer types, comorbidity burden (aCCI), functional status (ADL), and illness severity (qSOFA)—did not differ significantly between MDR-KP and non-MDR-KP groups, nor between ESBL-KP and non-resistant groups (**Table 2**; **Supplementary Table S3**). Recent hospitalization and urinary catheterization were more frequent in both resistant-phenotype groups (all *P* < 0.05). Additional factors were observed specifically in the MDR-KP group, including higher rates of ventilator use and recent surgery. Laboratory findings also indicated a more pronounced inflammatory response in MDR-KP patients, reflected by elevated WBC, Neu, SII, and MLR (all *P* < 0.05) (**Table 2**). In contrast, the ESBL-KP group showed limited significant laboratory differences, with only platelet (PLT) count (*P* = 0.028) and total bilirubin (TB) level (*P* = 0.029) reaching statistical significance (**Supplementary Table 3**). Regarding economic impact, antibiotic expenditures were substantially higher in both the MDR-KP (*P* < 0.001) and ESBL-KP (*P* = 0.007) groups compared to their non-resistant counterparts, though total hospitalization costs and overall medical fees did not differ significantly (**Table 2**; **Supplementary Table 3**).

**Table 2:** Comparison of clinical characteristics between MDR-KP and non-MDR-KP groups.

Variables	Total (n=153)	MDR-KP(n=60)	non-MDR-KP(n=93)	*P* value*
Sex, M, No. (%)	106 (69.3)	37 (61.7)	69 (74.2)	0.101
Age, y, median (IOR)	68 (58, 74)	68 (57, 74)	68 (58, 74)	0.693
BMI, median (IQR)	20.76 (18.39, 24.23)	21.66 (19.03, 24.48)	20.76 (18.08, 23.95)	0.392
History, No. (%)				
Drinking	41 (26.8)	15 (25.0)	26 (28.0)	0.687
Smoking	51 (33.3)	20 (33.3)	31 (33.3)	1.000
Hospitalization	104 (68.0)	47 (78.3)	57 (61.3)	0.027
Infection	52 (34.0)	22 (36.7)	30 (32.3)	0.574
Infection type, No. (%)				
Community acquired	53 (34.6)	19 (31.7)	34 (36.6)	0.535
Healthcare associated	72 (47.1)	32 (53.3)	40 (43.0)	0.212
Hospital acquired	28 (18.3)	9 (15.0)	19 (20.4)	0.396
Cancer type, No. (%)				
Lung cancer	32 (20.9)	12 (20.0)	20 (21.5)	0.823
Gastrointestinal cancer	47 (30.7)	15 (25.0)	32 (34.4)	0.218
Hepatobiliary and Pancreatic cancer	31 (20.3)	13 (21.7)	18 (19.4)	0.728
Others	43 (28.1)	20 (33.3)	23 (24.7)	0.248
With metastasis	86 (56.2)	28 (46.7)	58 (62.4)	0.056
Comorbid conditions, No. (%)				
Hypertension	57 (37.3)	24 (40.0)	33 (35.5)	0.573
Diabetes mellitus	32 (20.9)	14 (23.3)	18 (19.4)	0.555
Nervous system diseases	34 (22.2)	18 (30.0)	16 (17.2)	0.063
Respiratory diseases	34 (22.2)	16 (26.7)	18 (19.4)	0.288
Cardiovascular diseases	31 (20.3)	13 (21.7)	18 (19.4)	0.728
Digestive diseases	21 (13.7)	5 (8.3)	16 (17.2)	0.120
Chronic kidney diseases	20 (13.1)	10 (16.9)	10 (10.8)	0.289
Chronic hepatic diseases	36 (23.5)	14 (23.3)	22 (23.7)	0.963
**Hypoproteinemia, No (%)**	88 (57.5)	39 (65.0)	49 (52.7)	0.133
**aCCI, median (IQR)**	8 (6, 9)	7 (5, 9)	8 (6, 9)	0.118
**ADL score at admission, median (IQR)**	95 (78, 100)	95 (70, 100)	95 (80, 100)	0.454
**ADL score at discharge, median (IQR)**	90 (70, 100)	90 (56, 100)	95 (80, 100)	0.119
**qSOFA at admission ≥1, No. (%)**	45 (29.4)	22 (36.7)	23 (24.7)	0.114
Invasive procedure, No. (%)				
Urinary catheter	33 (21.6%)	20 (33.3%)	13 (14.0%)	0.004
Percutaneous catheter drainage	59 (38.6%)	23 (38.3%)	36 (38.7%)	0.963
Venous and arterial catheter	55 (35.9%)	26 (43.3%)	29 (31.2%)	0.126
Use of ventilators	20 (13.1%)	12 (20.0%)	8 (8.6%)	0.041
Tracheal cannula	21 (13.7%)	10 (16.7%)	11 (11.8%)	0.396
Endoscope	30 (19.6%)	8 (13.3%)	22 (23.7%)	0.116
Others	10 (6.5%)	6 (10.0%)	4 (4.3%)	0.290
Invasive procedure within 7 d	56 (36.6%)	25 (41.7%)	31 (33.3%)	0.296
Surgery within 90 d	49 (32.0%)	26 (43.3%)	23 (24.7%)	0.016
Laboratory findings at admission, median (IQR)				
WBC, ×10^9/L	6.52 (4.42, 10.01)	8.11 (4.91, 11.95)	5.96 (4.20, 9.32)	0.008
Neu, ×10^9/L	4.85 (2.98. 8.31)	6.43 (3.47, 10.88)	4.16 (2.76, 7.16)	0.006
Lym, ×10^9/L	0.78 (0.45, 1.36)	0.78 (0.44, 1.38)	0.77 (0.46, 1.36)	0.828
Mon, ×10^9/L	0.39 (0.24, 0.57)	0.42 (0.27, 0.66)	0.33 (0.20, 0.56)	0.075
PLT, ×10^9/L	151 (111, 223)	179 (123, 240)	143 (98, 207)	0.087
Hb, g/L, (mean ± SD)	107 ± 21	105 ± 22	108 ± 21	0.306
SII	960.54 (450.34, 1956.42)	1376.41 (528.99, 2220.32)	768.45 (378.76, 1777.93)	0.013
NLR	6.50 (2.93, 11.44)	8.07 (3.88, 15.29)	6.15 (2.57, 10.74)	0.071
PLR	203.17 (123.72, 318.47)	214.07 (133.04, 340.92)	188.33 (119.76, 284.45)	0.221
MLR	0.48 (0.27, 0.80)	0.59 (0.34,.93)	0.46 (0.24, 0.68)	0.040
ALT, U/L	18.6 (12.0, 30.7)	19.6 (13.0, 36.0)	18.0 (11.7, 30.2)	0.408
AST, U/L	26.8 (18.1, 43.2)	19.4 (18.2, 39.8)	26.4 (18.1, 45.3)	0.514
TB, μmol/L	14.03 (9.84, 20.60)	12.17 (8.73, 20.27)	14.74 (10.47, 21.50)	0.085
TP, g/L	60.1 (55.7, 65.4)	61.5 (56.3, 65.3)	59.9 (55.3, 65.5)	0.634
ALB, g/L	34.7 (30.8, 38.4)	34.0 (30.5, 36.9)	35.2 (31.4, 39.7)	0.102
Cr, μmol/L	62.4 (50.9, 79.1)	63.2 (51.1, 84.5)	60.0 (50.7, 77.8)	0.559
Bun, mmol/L	5.14 (4.25, 7.55)	5.68 (4.17, 9.40)	5.05 (4.28, 7.08)	0.241
**Fever, No. (%)**	64 (41.8)	27 (45.0)	37 (39.8)	0.523
Fever ≥ 72h	35 (22.8)	16 (26.7)	19 (20.4)	0.370
**Bacteremia, No. (%)**	16 (10.5)	5 (8.3)	11 (11.8)	0.490
**Shock, No. (%)**	8 (5.2)	3 (5.0)	5 (5.4)	1.000
Related cost(dollars)^#^, median (IQR)				
Total cost	2796.6 (1603.6, 6628.5)	3081.8 (1616.3, 9601.2)	2560.4 (1524.3, 6298.4)	0.245
Medical fee	1216.8 (583.5, 2167.4)	1270.2 (590.7, 2925.8)	1109.9 (580.3, 1801.1)	0.207
Antibacterial agent cost	44.3 (8.4, 220.9)	583.7 (26.4, 292.2)	20.4 (2.9, 138.7)	<0.001
Treatment with antibiotics, No. (%)				
Empirical antibiotic therapy	125 (81.7)	52 (86.7)	73 (78.5)	0.202
Second/third-generation cephalosporins	53 (34.6)	17 (28.3)	36 (38.7)	0.188
β-lactam/β-lactamase Inhibitor	62 (40.5)	32 (53.3)	30 (32.3)	**0.010**
Carbapenems	19 (12.4)	10 (16.7)	9 (9.7)	0.201
Quinolones	16 (10.5)	5 (8.3)	11 (11.8)	0.490
Aminoglycosides	7 (4.6)	5 (8.3)	2 (2.2)	0.164
Nitroimidazoles	11 (7.2)	5 (8.3)	6 (6.5)	0.905
Others	7 (4.6)	3 (5.0)	4 (4.3)	1.000
Single DAT, No. (%)				
Second/third-generation cephalosporins	46 (30.1)	17 (28.3)	29 (31.2)	0.707
β-lactam/β-lactamase Inhibitor	48 (31.4)	21 (35.0)	27 (29.0)	0.437
Carbapenems	19 (12.4)	9 (15.0)	10 (10.8)	0.437
Quinolones	21 (13.7)	5 (8.3)	16 (17.2)	0.120
Aminoglycosides	8 (5.2)	7 (11.7)	1 (1.1)	**0.012**
Others	4 (2.6)	3 (5.0)	1 (1.1)	0.334
Combined DAT, No. (%)				
Combination of two antimicrobials	36 (23.5)	20 (33.3)	16 (17.2)	**0.022**
Combination of ≥ triple antimicrobials	4 (2.6)	4 (6.7)	0 (0)	**0.045**
Tumor treatment, No. (%)				
Radiotherapy	27 (17.6)	10 (16.7)	17 (18.3)	0.798
Chemotherapy	96 (62.7)	33 (55.0)	63 (67.7)	0.111
Immunotherapy	50 (32.7)	22 (36.7)	28 (30.1)	0.398
Targeted therapy	73 (47.7)	29 (48.3)	44 (47.3)	0.902
Herbal therapy	40 (26.1)	15 (25.0)	25 (26.9)	0.796
Concomitant drugs, No. (%)				
Immunosuppressant	121 (79.1)	52 (86.7)	69 (74.2)	0.064
Hormone	130 (85.0)	55 (91.7)	75 (80.6)	0.063
Clinical outcomes				
LOS in ICU, median (IQR)	0 (0, 0)	0 (0, 0)	0 (0, 0)	0.077
ICU, No. (%)	13 (8.5)	8 (13.3)	5 (5.4)	0.085
LOS, median (IQR)	12 (9, 23)	13 (9, 32)	12 (8, 20)	0.139
LOS after infection	9 (5, 16)	11 (6, 12)	9 (5, 16)	0.199
Clinical stability, No. (%)	122 (79.7)	46 (76.7)	76 (81.7)	0.448
On the mend, No. (%)	9 (5.9)	4 (6.7)	5 (5.4)	1.000
Deterioration, No. (%)	13 (8.5)	6 (10.0)	7 (7.5)	0.592
Automatic discharge or transfer, No. (%)	26 (17.0)	12 (20.0)	14 (15.1)	0.426
Readmission, No. (%)	97 (63.4)	37 (61.7)	60 (64.5)	0.721
Reinfection, No. (%)	27 (17.6)	10 (16.7)	17 (18.3)	0.798
Palliative care, No. (%)	11 (7.2)	3 (5.0)	8 (8.6)	0.602

*P values are obtained using chi-square test or fisher’s exact test for categorical variables and the independent-samples t-test or Mann-Whitney *U* for continuous variables. ^#^1 dollar = 7.1138 Chinese Yuan as of September, 2025. Data are represented as No. (%), IQR and mean ± SD. Bold values indicate statistical significance (*P* < 0.05).

Mon, monocytes; Cr, creatinine; Bun, blood urea nitrogen.

9 patients had CRKP infection (median age, 71 years; 7 men and 2 women). 3 infections involved the respiratory tract, 2 the bloodstream, 2 the abdomen, and 2 the urinary tract. 4 patients required ICU care. 5 isolates met the MDR definition, 3 were XDR, and 1 was PDR. 6 patients were readmitted; 2 experienced clinical deterioration and discharge against medical advice or transfer, and no in-hospital deaths occurred. Patient-level characteristics and outcomes are reported in **Supplementary Table 4**.

### 3.3 Site-specific and cancer-type-specific characteristics of KP infection

**Figure 2:**
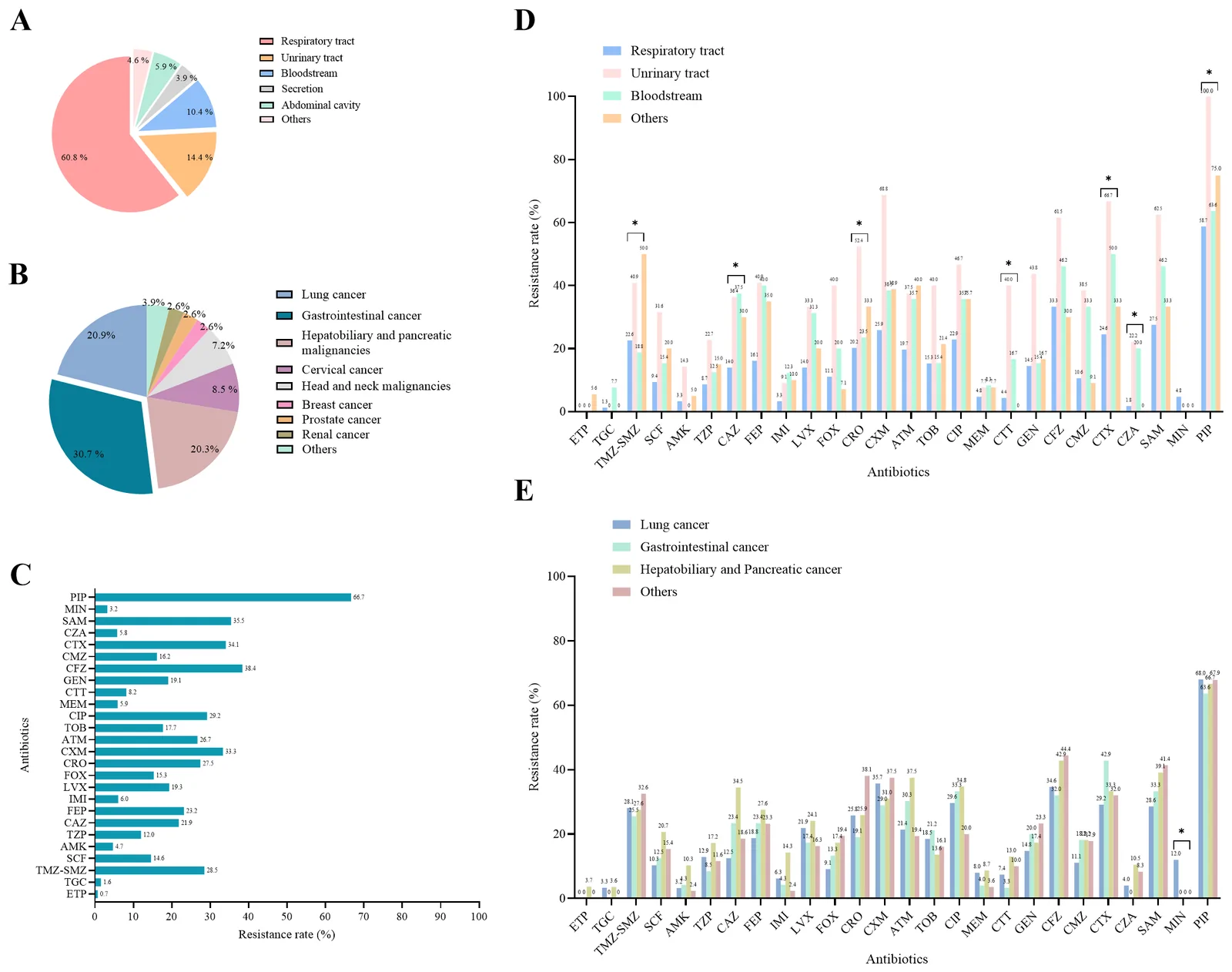
Microbiological characteristics of the 153 KP isolates included. **(A)** Distribution by infection site. **(B)** Distribution by cancer type. **(C)** Overall resistance rates. **(D)** Resistance rates by infection site. **(E)** Resistance rates by cancer type. *P* values were obtained using the chi-square test or Fisher’s exact test. Statistical significance between the two groups is denoted as *P < 0.05. Resistance rates were calculated based on the number of isolates tested for each antimicrobial agent because not all antimicrobial agents were routinely tested for every isolate (**Supplementary Table 1**). ETP, ertapenem; TGC, tigecycline; TMP-SMX; trimethoprim-sulfamethoxazole; SCF, cefoperazone-sulbactam; AMK, amikacin; TZP, piperacillin-tazobactam; CAZ, ceftazidime; FEP, cefepime; IMI, imipenem; LVX, levofloxacin; FOX, cefoxitin; CRO, ceftriaxone; CXM, cefuroxime; ATM, aztreonam; TOB, tobramycin; CIP, ciprofloxacin; MEM, meropenem; CTT, cefotetan; GEN, gentamicin; CFZ, cefazolin; CMZ, cefmetazole; CTX, cefotaxime; CZA, ceftazidime-avibactam; SAM, ampicillin-sulbactam; MIN, minocycline; PIP, piperacillin.

Analysis of 153 KP isolates revealed the respiratory tract as the most common infection site (93/153, 60.8%), followed by the urinary tract (22/153, 14.4%) and bloodstream (16/153, 10.4%). KP infections were most frequently documented in patients with gastrointestinal cancer (47/153, 30.7%) and lung cancer (32/153, 20.9%). Antimicrobial susceptibility testing against 26 agents showed the highest overall resistance to piperacillin (66.7%) and the lowest to ertapenem (0.7%) (**Figures 2A–C**).

Urinary tract isolates had the highest reported proportions of MDR-KP (69.6%, *P* = 0.005) and ESBL-KP (60.9%, *P* = 0.016) (**Table 3**). As illustrated in **Figure 2D**, these isolates demonstrated universal resistance to piperacillin (100%) and a high resistance burden (> 60%) against several cephalosporins and beta-lactamase inhibitor combinations, including cefuroxime (68.8%), cefotaxime (66.7%), cefazolin (61.5%), and ampicillin-sulbactam (62.5%). In contrast, patients with BSI presented more pronounced with the most severe clinical and laboratory profiles (**Table 3**), showing significantly higher inflammatory and tissue injury markers—including elevated Neu count, NLR, alanine aminotransferase (ALT), aspartate aminotransferase (AST), and TB (all *P* < 0.005). This group also had the lowest median Lym count (*P* = 0.003) and ALB level (*P* = 0.001). Clinically, BSI patients experienced the highest rates of fever (*P* = 0.039), bacteremia (*P* < 0.001), and shock (*P* = 0.017), the lowest rate of clinical stability at discharge (*P* = 0.020), and the highest median antibiotic costs (*P* = 0.005). Respiratory tract isolates were generally associated with lower resistance rates (**Figure 2D**). In contrast, infections at other sites were more frequently accompanied by invasive procedures, including puncture drainage (*P* = 0.002) and recent surgery (*P* = 0.06), and higher antibiotic expenditures (*P* = 0.005) (**Table 3**).

**Table 3:** Comparison of clinical features across different sites of separation.

Variables	Total (n=153)	Respiratory tract (n=93)	Urinary tract (n=22)	Bloodstream (n=16)	Others (n=22)	*P* value*
**Sex, M, No. (%)**	106 (69.3)	77 (82.8)	8 (34.8)	7 (43.8)	14 (66.7)	**<0.001*^bc^***
**Age, y, median (IQR)**	68 (58, 74)	69 (57, 75)	61 (56, 70)	61 (56, 74)	71 (60, 75)	0.146
**BMI, (mean± SD)**	21.49 ± 3.96	21.93 ± 4.18	21.33 ± 3.55	19.90 ± 3.26	19.21 ± 3.70	0.237
**MDR-KP, No. (%)**	60 (39.2)	28 (30.1)	16 (69.6)	6 (37.5)	10 (47.6)	**0.005*^b^***
**ESBL-KP, No. (%)**	51 (33.3)	25 (26.9)	14 (60.9)	4 (25.0)	8 (38.1)	**0.016*^b^***
**CRKP, No. (%)**	9 (5.9)	3 (3.2)	2 (8.7)	2 (12.5)	2 (9.5)	0.377
History, No. (%)						
Drinking	41 (26.8)	33 (35.5)	2 (8.7)	1 (6.3)	5 (23.8)	**0.005*^a^***
Smoking	51 (33.3)	37 (39.8)	3 (13.0)	5 (31.3)	6 (28.6)	0.100
Hospitalization	104 (68.0)	61 (65.6)	17 (73.9)	14 (87.5%	12 (57.1)	0.208
Infection	52 (34.0)	27 (29.0)	10 (43.5)	7 (43.8)	8 (38.1)	0.427
Infection type, No. (%)						
Community acquired	53 (34.6)	37 (39.8)	7 (30.4)	4 (25.0)	5 (23.8)	0.390
Healthcare associated	72 (47.1)	36 (38.7)	12 (52.2)	10 (62.5)	14 (66.7)	0.055
Hospital acquired	28 (18.3)	20 (21.5)	4 (17.4)	2 (12.5)	2 (9.5)	0.515
Tumor type, No. (%)						
Lung cancer	32 (20.9)	32 (34.4)	0 (0)	0 (0)	0 (0)	**<0.001*^bcd^***
Gastrointestinal cancer	47 (30.7)	30 (32.3)	7 (30.4)	4 (25.0)	6 (28.6)	0.939
Hepatobiliary and Pancreatic cancer	31 (20.3)	10 (10.8)	2 (8.7)	9 (56.3)	10 (47.6)	**<0.001*^cedf^***
Others	43 (28.1)	21 (22.6)	14 (60.9)	3 (18.8)	5 (23.8)	**0.004*^b^***
With metastasis	86 (56.2)	57 (61.3)	12 (52.2)	5 (31.3)	12 (57.1)	0.159
Comorbid conditions, No. (%)						
Hypertension	57 (37.3)	31 (33.3)	9 (39.1)	5 (31.3)	12 (57.1)	0.217
Diabetes mellitus	32 (20.9)	17 (18.3)	3 (13.0)	2 (12.5)	10 (47.6)	**0.023*^d^***
Nervous system diseases	34 (22.2)	18 (19.4)	7 (30.4)	3 (18.8)	6 (28.6)	0.601
Respiratory diseases	34 (22.2)	24 (25.8)	2 (8.7)	2 (12.5)	6 (28.6)	0.164
Cardiovascular diseases	31 (20.3)	18 (19.4)	3 (13.0)	3 (18.8)	7 (33.3)	0.415
Digestive diseases	21 (13.7)	13 (14.0)	2 (8.7)	4 (25.0)	2 (9.5)	0.505
Chronic kidney diseases	20 (13.1)	11 (11.8)	4 (17.4)	2 (12.5)	3 (14.3)	0.917
Chronic hepatic diseases	36 (23.5)	13 (14.0)	6 (26.1)	8 (50.0)	9 (42.9)	**0.002*^cd^***
**Hypoproteinemia, No. (%)**	88 (57.5)	46 (49.5)	10 (43.5)	16 (100.0)	16 (76.2)	**<0.001*^ce^***
**aCCI, median (IQR)**	8 (6, 9)	8 (6, 9)	6 (4, 9)	6 (4, 9)	7 (6, 10)	**0.043*^a^***
**ADL score at admission, median (IQR)**	95 (78, 100)	95 (75, 100)	100 (90, 100)	90 (73, 100)	90 (68, 98)	**0.045 *^a^***
**ADL score at discharge, median (IQR)**	90 (70, 100)	90 (70, 100)	100 (95, 100)	85 (24, 90)	80 (58, 95)	**<0.001*^ef^***
**qSOFA at admission ≥ 1 , No. (%)**	45 (29.4)	25 (26.9)	7 (30.4)	6 (37.5)	7 (33.3)	0.817
Invasive procedure, No. (%)						
Urinary catheter	33 (21.6)	14 (15.1)	10 (43.5)	4 (25.0)	5 (23.8)	**0.042*^b^***
Percutaneous catheter drainage	59 (38.6)	27 (29.0)	8 (34.8)	10 (62.5)	14 (66.7)	**0.002*^d^***
Venous and arterial catheter	55 (35.9)	31 (33.3)	5 (21.7)	6 (37.5)	13 (61.9)	**0.037*^f^***
Use of ventilators	20 (13.1)	9 (9.7)	3 (13.0)	3 (18.8)	5 (23.8)	0.361
Tracheal cannula	21 (13.7)	12 (12.9)	2 (8.7)	3 (18.8)	4 (19.0)	0.711
Endoscope	30 (19.6)	23 (24.7)	1 (4.3)	4 (25.0)	2 (9.5)	**0.047*^a^***
Others	10 (6.5)	3 (3.2)	3 (13.0)	3 (18.8)	1 (4.8)	0.107
Invasive procedure within 7 d	56 (36.6)	35 (37.6)	6 (26.1)	8 (50.0)	7 (33.3)	0.480
Surgery within 90 d	49 (32.0)	20 (21.5)	10 (43.5)	8 (50.0)	11 (52.4)	**0.006*^d^***
Laboratory findings following infection, median (IQR)						
WBC, ×10^9/L	6.52 (4.42, 10.01)	6.48 (4.49, 9.45)	5.39 (4.13, 8.67)	8.67 (4.00, 14.16)	9.74 (5.53, 11.74)	0.148
Neu, ×10^9/L	4.85 (2.98. 8.31)	4.48 (2.91, 7.74)	3.42 (2.47, 6.80)	7.95 (3.30, 12.62)	6.34 (3.81, 10.13)	**0.043*^a^***
Lym, ×10^9/L	0.78 (0.45, 1.36)	0.79 (0.49, 1.39)	1.01 (0.63, 1.45)	0.36 (0.27, 0.77)	0.84 (0.48, 1.76)	**0.003*^ceg^***
Mon, ×10^9/L	0.39 (0.24, 0.57)	0.39 (0.26, 0.57)	0.40 (0.13, 0.55)	0.25 (0.14, 0.43)	0.45 (0.30, 0.70)	0.074
PLT, ×10^9/L	151 (111, 223)	144 (116, 222)	175 (128, 229)	110 (56, 194)	168 (122, 253)	0.096
Hb, g/L	104 (92, 121)	108 (95, 124)	106 (92, 118)	98 (86, 119)	99 (88, 108)	0.102
SII	960.54 (450.34, 1956.42)	887.80 (435.16, 1891.90)	609.57 (322.09, 1738.67)	1518.33 (538.98, 2921.00)	1310.59 (554.98, 1949.07)	0.221
NLR	6.50 (2.93, 11.44)	6.22 (2.93, 10.74)	3.90 (2.27, 9.79)	11.10 (8.42, 32.83)	8.45 (3.46, 12.86)	**0.003*^ce^***
PLR	203.17 (123.72, 318.47)	195.24 (119.76, 290.55)	180.85 (136.36, 266.67)	248.57 (145.4, 588.54)	213.73 (126.80, 343.74)	0.460
MLR	0.48 (0.27, 0.80)	0.47 (0.27, 0.76)	0.33 (0.15, 0.66)	0.53 (0.36, 0.90)	0.57 (0.34, 0.94)	0.277
ALT, U/L	18.6 (12.0, 30.7)	17.1 (11.3, 28.8)	16.1 (9.8, 25.0)	30.8 (18.8, 89.2)	26 (14.9,36,6)	**0.005*^ce^***
AST, U/L	26.8 (18.1, 43.2)	25.8 (17.6, 37.8)	23 (17.5, 31.9)	55.6 (39.3, 197.1)	30.5 (18.7, 54.5)	**<0.001*^ceg^***
TB, μmol/L	14.03 (9.84, 20.60)	13.02 (9.49, 18.87)	10.84 (8.79, 17.36)	24.71 (19.82, 103.08)	18.16 (10.21, 26.81)	**<0.001*^ce^***
TP, g/L	60.1 (55.7, 65.4)	60.3 (55.7, 65.4)	63.3 (58.5, 72.0)	56.1 (54.0, 65.7)	58.9 (55.2, 64.8)	0.093
ALB, g/L	34.7 (30.8, 38.4)	35.3 (32, 39.3)	35.4 (33.0, 41.7)	30.6 (26.6, 37.3)	31.2 (27.2, 35.0)	**0.001*^df^***
Cr, μmol/L	62.4 (50.9, 79.1)	62.8 (51.35, 77.8)	57 (49.3, 99.8)	65.85 (43.8, 99.3)	61.8 (51.6, 80.5)	0.970
Bun, mmol/L	5.14 (4.25, 7.55)	5.22 (4.32, 7.54)	4.98 (3.70, 7.14)	7.61 (4.51, 14.88)	5.05 (3.86, 6.94)	0.225
**Fever, No. (%)**	64 (41.8)	35 (37.6)	8 (34.8)	12 (75.0)	9 (42.9)	**0.039*^c^***
Fever ≥ 72h	35 (22.8)	20 (21.5)	4 (17.4)	7 (43.8)	4 (19.0)	0.251
**Bacteremia, No. (%)**	16 (10.5)	1 (1.1)	0 (0)	14 (87.5)	1 (4.8)	**<0.001*^ceg^***
**Shock, No. (%)**	8 (5.2)	3 (3.2)	0 (0)	4 (25.0)	1 (4.8)	**0.017*^c^***
Related cost(dollars)^#^, median (IQR)						
Total cost	2796.6 (1603.6, 6628.5)	2435.3 (1404.3, 6622.3)	2801.1 (1263.8, 6164.0)	5243.7 (2687.2, 11771.9)	2861.3 (1975.3, 5845.1)	0.102
Medical fee	1216.8 (583.5, 2167.4)	1075.9 (549.9, 1906.5)	1033.3 (434.7, 1776.8)	1777.7 (1000.3, 5511.74)	1277.9 (833.0, 3973.9)	0.070
Antibacterial agent cost	44.3 (8.4, 220.9)	23.8 (4.2, 151.4)	55.9 (22.2, 112.4)	203.8 (30.7, 414.8)	210.1 (21.6, 327.1)	**0.005*^d^***
Treatment with antibiotics, No. (%)						
Empirical antibiotic therapy	125 (81.7)	72 (77.4)	17 (73.9)	16 (100.0)	20 (95.2)	**0.008*^a^***
Second/third-generation cephalosporins	53 (34.6)	34 (36.6)	10 (43.5)	3 (18.8)	6 (28.6)	0.381
β-lactam/β-lactamase Inhibitor	62 (40.5)	32 (34.4)	4 (17.4)	11 (68.8)	15 (71.4)	**<0.001*^def^***
Carbapenems	19 (12.4)	6 (6.5)	3 (13.0)	7 (43.8)	3 (14.3)	**0.004*^c^***
Quinolones	16 (10.5)	12 (12.9)	4 (17.4)	0 (0)	0 (0)	**0.021*^a^***
Aminoglycosides	7 (4.6)	2 (2.2)	1 (4.3)	3 (18.8)	1 (4.8)	0.120
Nitroimidazoles	11 (7.2)	2 (2.2)	0 (0)	3 (18.8)	6 (28.6)	**<0.001*^cdf^***
Others	7 (4.6)	4 (4.3)	0 (0)	2 (12.5)	1 (4.8)	0.288
Single DAT, No. (%)						
Second/third-generation cephalosporins	46 (30.1)	29 (31.2)	11 (47.8)	1 (6.3)	5 (23.8)	0.026
β-lactam/β-lactamase Inhibitor	48 (31.4)	23 (24.7)	6 (26.1)	9 (56.3)	10 (47.6)	**0.025*^a^***
Carbapenems	19 (12.4)	5 (5.4)	4 (17.4)	5 (31.3)	5 (23.8)	**0.009*^cd^***
Quinolones	21 (13.7)	16 (17.2)	3 (13.0)	1 (6.3)	1 (4.8)	0.301
Aminoglycosides	8 (5.2)	4 (4.3)	2 (8.7)	1 (6.3)	1 (4.8)	0.877
Others	4 (2.6)	2 (2.2)	0 (0)	0 (0)	0 (0)	0.128
Combined DAT, No. (%)						
Combination of two antimicrobials	36 (23.5)	20 (21.5)	3 (13.0)	6 (37.5)	7 (33.3)	0.221
Combination of ≥ triple antimicrobials	4 (2.6)	1 (1.1)	0 (0)	1 (6.3)	2 (9.5)	0.151
Tumor treatment, No. (%)						
Radiotherapy	27 (17.6)	20 (21.5)	3 (13.0)	2 (12.5)	2 (9.5)	0.442
Chemotherapy	96 (62.7)	63 (67.7)	15 (65.2)	9 (56.3)	9 (38.1)	0.180
Immunotherapy	50 (32.7)	29 (31.2)	9 (39.1)	6 (37.5)	6 (28.6)	0.835
Targeted therapy	73 (47.7)	51 (54.8)	7 (40.4)	6 (37.5)	9 (42.9)	0.138
Herbal therapy	40 (26.1)	28 (30.1)	5 (21.7)	1 (6.3)	6 (28.6)	0.149
Concomitant drugs, No. (%)						
Immunosuppressant	121 (79.1)	74 (79.6)	20 (87.0)	12 (75.0)	15 (71.45)	0.611
Hormone	130 (85.0)	76 (81.7)	21 (91.3)	14 (87.5)	19 (90.5)	0.531
Clinical outcomes						
LOS in ICU, median (IQR)	0 (0, 0)	0 (0, 0)	0 (0, 0)	0 (0, 0)	0 (0, 0)	0.211
ICU, No. (%)	13 (8.5)	9 (9.7)	0 (0)	3 (18.8)	1 (4.8)	0.096
LOS, median (IQR)	12 (9, 23)	12 (8, 23)	11 (8, 16)	16 (11, 37)	14 (11, 25)	0.415
LOS after infection	9 (5, 16)	8 (5, 16)	10 (4, 14)	11 (7, 24)	13 (10, 18)	0.105
Clinical stability, No. (%)	122 (79.7)	78 (83.9)	21 (91.3)	9 (56.3)	14 (66.7)	**0.020*^a^***
On the mend, No. (%)	9 (5.9)	3 (3.2)	2 (8.7)	2 (12.5)	2 (9.5)	0.377
Deterioration, No. (%)	13 (8.5)	6 (6.5)	0 (0)	4 (25.0)	3 (14.3)	**0.026*^a^***
Automatic discharge or transfer, No. (%)	26 (17.0)	14 (15.1)	1 (4.3)	5 (31.3)	6 (28.6)	0.059
Readmission, No. (%)	97 (63.4)	67 (72.0)	15 (65.2)	5 (31.3)	10 (47.6)	**0.006*^a^***
Reinfection, No. (%)	27 (17.6)	17 (18.3)	2 (8.7)	3 (18.8)	5 (23.8)	0.563
Palliative care, No. (%)	11 (7.2)	7 (7.5)	1 (4.3)	2 (12.5)	1 (4.8)	0.775

*P values are obtained using the chi-square for categorical variables and Kruskal-Wallis test for continuous variables. ^#^1 dollar = 7.1138 Chinese Yuan as of September, 2025. Data are represented as No. (%), IQR and mean ± SD. Bold values indicate statistical significance (*P* < 0.05).

^a^No significant difference between any of the pairs in *post hoc* analysis.

^b^Significantly different between respiratory tract and urinary tract groups.

^c^Significantly different between respiratory tract and bloodstream groups.

^d^Significantly different between respiratory tract and others groups.

^e^Significantly different between urinary tract and bloodstream groups.

^f^Significantly different between urinary tract and others groups. g: Significantly different between bloodstream and others groups.

Mon, monocytes; Cr, creatinine; Bun, blood urea nitrogen.

Stratification by cancer type revealed distinct resistance profiles and clinical severity (**Figure 2E**). KP isolates obtained from patients with lung cancer showed the highest resistance to minocycline (12.0%) and piperacillin (68.0%), whereas isolates from gastrointestinal cancer patients showed the highest resistance to tobramycin (21.2%) and cefotaxime (42.9%). Isolates from patients with hepatobiliary and pancreatic cancer showed higher resistance to several agents. Clinically, patients with lung cancer had higher BMI, ALB, and Hb, and lower NLR, hypoproteinemia and bacteremia frequencies (all *P* < 0.05) (**Table 4**). Conversely, patients with hepatobiliary and pancreatic cancers exhibited more severe disease manifestations, including elevated inflammatory markers (WBC and Neu; *P* < 0.01), more pronounced hepatic injury (ALT, AST, and TB; all *P* < 0.001), and a higher prevalence of hypoproteinemia and chronic liver disease (both *P* < 0.001). Gastrointestinal cancer patients demonstrated intermediate severity but had the highest NLR and proportion of HAI. Treatment patterns also differed, with hepatobiliary and pancreatic cancer patients receiving the most β-lactam/β-lactamase inhibitor therapy (*P* = 0.005) and incurring the highest antibacterial costs (*P* = 0.043), whereas lung cancer patients had the highest readmission rates (*P* = 0.031) (**Table 4**).

**Table 4:** Comparison of clinical features across different cancerous tumors.

Variables	Total (n=153)	Lung cancer (n=32)	Gastrointestinal cancer (n=47)	Hepatobiliary and pancreatic cancer (n=31)	Others (n=43)	*P* value*
**Sex, M, No. (%)**	106 (69.3)	25 (78.1)	40 (85.1)	17 (54.8)	24 (55.5)	**0.004*^ef^***
**Age, y, (mean ± SD)**	66 ± 11	66 ± 10	69 ± 11	65 ± 11	63 ± 11	0.112
**BMI, median (IQR)**	20.76 (18.39, 24.23)	22.96 (20.73, 25.69)	19.22 (17.51, 22.53)	19.47 (18.13, 21.30)	22.94 (19.92, 24.56)	**<0.001 *^bcfg^***
**MDR-KP, No. (%)**	60 (39.2)	12 (37.5)	15 (31.9)	13 (41.9)	20 (46.5)	0.542
**ESBL-KP, No. (%)**	51 (33.3)	10 (31.3)	13 (27.7)	9 (29.0)	19 (44.2)	0.350
**CRKP, No. (%)**	9 (5.9)	2 (6.3)	2 (4.3)	4 (12.9)	1 (2.3)	0.306
History, No. (%)						
Drinking	41 (26.8)	9 (28.1)	17 (36.2)	6 (19.4)	9 (20.9)	0.288
Smoking	51 (33.3)	12 (37.5)	21 (44.7)	7 (22.6)	11 (25.6)	0.124
Hospitalization	104 (68.0)	21 (65.6)	29 (61.7)	22 (71.0)	32 (74.4%	0.598
Infection	52 (34.0)	11 (34.4)	15 (31.9)	12 (38.7)	14 (32.6)	0.932
Infection type, No. (%)						
Community acquired	53 (34.6)	19 (59.4)	16 (34.0)	10 (32.3)	8 (18.6)	**0.003*^d^***
Healthcare associated	72 (47.1)	12 (37.5)	19 (40.4)	15 (48.4)	26 (60.5)	0.163
Hospital acquired	28 (18.3)	1 (3.1)	12 (25.5)	6 (19.4)	9 (20.9)	0.079
Comorbid conditions, No. (%)						
Hypertension	57 (37.3)	14 (43.8)	19 (40.4)	12 (38.7)	12 (16.0)	0.491
Diabetes mellitus	32 (20.9)	8 (25.0)	9 (19.1)	8 (25.8)	7 (16.3)	0.701
Nervous system diseases	34 (22.2)	6 (18.8)	9 (19.1)	9 (29.0)	10 (23.3)	0.720
Respiratory diseases	34 (22.2)	14 (43.8)	10 (21.3)	5 (16.1)	5 (11.6)	**0.007*^d^***
Cardiovascular diseases	31 (20.3)	9 (28.1)	12 (25.5)	5 (16.1)	5 (11.6)	0.227
Digestive diseases	21 (13.7)	0 (0)	13 (27.7)	4 (12.9)	4 (9.3)	**<0.001*^b^***
Chronic kidney diseases	20 (13.1)	4 (12.5)	6 (12.8)	5 (16.1)	5 (11.6)	0.953
Chronic hepatic diseases	36 (23.5)	4 (12.5)	7 (14.9)	17 (54.8)	8 (18.6)	**<0.001*^ceg^***
**Hypoproteinemia, No. (%)**	88 (57.5)	12 (37.5)	28 (59.6)	27 (87.1)	21 (48.8)	**<0.001*^cg^***
**aCCI, median (IQR)**	8 (6, 9)	8 ± 2	8 ± 2	7 ± 3	7 ± 2	**0.003*^f^***
**ADL score at admission, median (IQR)**	95 (78, 100)	95 (86, 100)	95 (70, 100)	90 (80, 100)	95 (80, 100)	0.437
**ADL score at discharge, median (IQR)**	90 (70, 100)	98 (75, 100)	90 (65, 100)	85 (70, 95)	95 (85, 100)	**0.030 *^a^***
**qSOFA at admission ≥1, No. (%)**	45 (29.4)	9 (28.1)	16 (34.0)	12 (38.7)	8 (18.6)	0.239
Invasive procedure, No. (%)						
Urinary catheter	33 (21.6)	5 (15.6)	9 (19.1)	5 (16.1)	14 (32.6)	0.217
Percutaneous catheter drainage	59 (38.6)	9 (28.1)	12 (25.5)	22 (71.0)	16 (37.2)	**<0.001*^ceg^***
Venous and arterial catheter	55 (35.9)	10 (31.3)	14 (29.8)	16 (51.6)	15 (34.9)	0.221
Use of ventilators	20 (13.1)	5 (15.6)	5 (10.6)	2 (6.5)	8 (18.6)	0.406
Tracheal cannula	21 (13.7)	5 (15.6)	4 (8.5)	4 (12.9)	8 (18.6)	0.546
Endoscope	30 (19.6)	7 (21.9)	14 (29.8)	3 (9.7%)	6 (14.0)	0.111
Others	10 (6.5)	3 (9.4)	0 (0)	5 (16.1)	2 (4.7)	**0.015*^e^***
Invasive procedure within 7 d	56 (36.6)	12 (37.5)	13 (27.7)	15 (48.4)	16 (37.2)	0.322
Surgery within 90 d	49 (32.0)	5 (15.6)	13 (27.7)	13 (41.9)	18 (41.9)	0.053
Laboratory findings following infection, median (IQR)						
WBC, ×10^9/L	6.52 (4.42, 10.01)	6.79 (4.96, 9.75)	6.19 (3.99, 9.43)	9.49 (6.25, 15.20)	5.01 (3.49, 9.30)	**0.002*^eg^***
Neu, ×10^9/L	4.85 (2.98. 8.31)	4.46 (3.38, 7.19)	4.85 (3.24, 8.17)	8.24 (4.16, 13.93)	3.50 (2.12, 7.59)	**0.003*^eg^***
Lym, ×10^9/L	0.78 (0.45, 1.36)	1.27 (0.66, 1.54)	0.52 (0.39, 0.87)	0.84 (0.52, 1.35)	0.78 (0.47, 1.23)	**0.003*^b^***
Mon, ×10^9/L	0.39 (0.24, 0.57)	0.47 (0.30, 0.64)	0.32 (0.18, 0.52)	0.45 (0.27, 0.72)	0.32 (0.18, 0.50)	**0.006*^e^***
PLT, ×10^9/L	151 (111, 223)	179 (125, 263)	139 (111, 184)	157 (78, 244)	175 (120, 224)	0.189
Hb, g/L, (mean ± SD)	107 ± 21	116 ± 22	102 ± 19	106 ± 22	105 ± 20	**0.042*^b^***
SII	960.54 (450.34, 1956.42)	833.38 (370.43, 1563.24)	1270.70 (494.73, 2179.97)	1315.36 (422.73, 2617.19)	840.90 (454.81, 1750.41)	0.371
NLR	6.50 (2.93, 11.44)	4.53 (2.15, 8.38)	8.45 (4.26, 16.60)	9.08 (3.98, 15.91)	5.76 (2.85, 8.80)	**0.011*^b^***
PLR	203.17 (123.72, 318.47)	171.51 (93.31, 246.35)	240.00 (146.15, 358.33)	180.85 (108.28, 257.14)	213.73 (136.36, 318.99)	**0.029*^a^***
MLR	0.48 (0.27, 0.80)	0.45 (0.21, 0.68)	0.48 (0.30, 0.83)	0.61 (0.35, 0.91)	0.44 (0.23, 0.73)	0.282
ALT, U/L	18.6 (12.0, 30.7)	19.8 (12.2, 28.3)	17.4 (10.8, 28.0)	30.6 (24.3, 83.5)	14.1 (10.6, 20.0)	**<0.001*^ceg^***
AST, U/L	26.8 (18.1, 43.2)	24.4 (15.1, 30.4)	27.0 (18.1, 38.1)	59.1 (39.0, 139.0)	22.7 (17.0, 30.0)	**<0.001*^ceg^***
TB, μmol/L	14.03 (9.84, 20.60)	11.31 (8.28, 17.73)	14.39 (10.53, 19.60)	26.78 (19.34, 123.06)	10.73 (8.14, 15.18)	**<0.001*^ceg^***
TP, g/L	60.1 (55.7, 65.4)	64.1 (56.7, 69.3)	58.1 (54.2, 63.2)	58.0 (54.1, 67.1)	62.8 (57.8, 65.4)	**0.028*^a^***
ALB, g/L	34.7 (30.8, 38.4)	36.3 (33.7, 41.1)	34.2 (30.4, 37.3)	31.6 (27.0, 33.7)	35.1 (32.5, 40.2)	**<0.001*^cg^***
Cr, μmol/L	62.4 (50.9, 79.1)	64.2 (54.6, 77.4)	61.8 (51.2, 79.1)	57.3 (45.6, 73.0)	63.3 (51.5, 97.8)	0.402
Bun, mmol/L	5.14 (4.25, 7.55)	6.18 (4.62, 8.41)	5.23 (4.34, 7.31)	4.65 (3.20, 7.95)	5.14 (4.25, 7.14)	0.063
**Fever, No. (%)**	64 (41.8)	6 (18.8)	21 (44.7)	19 (61.3)	18 (41.9)	**0.007*^e^***
Fever ≥ 72h	35 (22.8)	5 (15.6)	10 (21.3)	10 (32.3)	10 (23.3)	0.462
**Bacteremia, No. (%)**	16 (10.5)	1 (3.1)	4 (8.5)	9 (29.0)	2 (4.7)	**0.005*^cg^***
**Shock, No. (%)**	8 (5.2)	1 (3.1)	4 (8.5)	2 (6.5)	1 (2.3)	0.532
Related cost(dollars)^#^, median (IQR)						
Total cost	2796.6 (1603.6, 6628.5)	2138.9 (1633.3, 6278.5)	2792.3 (1314.5, 7236.3)	3050.9 (2022.8, 11592.4)	2903.5 (1622.6, 6622.8)	0.422
Medical fee	1216.8 (583.5, 2167.4)	1047.8 (583.7, 2178.7)	1142.3 (458.1, 2379.8)	1609.6 (943.1, 4593.8)	1091.8 (532.9, 1816.3)	0.066
Antibacterial agent cost	44.3 (8.4, 220.9)	21.6 (0.5, 73.2)	29.8 (8.0, 213.2)	184.6 (22.6, 428.6)	42.6 (11.3, 460.3)	**0.043*^c^***
Treatment with antibiotics, No. (%)						
Empirical antibiotic therapy	125 (81.7)	24 (75.0)	42 (89.4)	29 (93.5)	30 (69.8)	**0.020*^a^***
Second/third-generation cephalosporins	53 (34.6)	9 (28.1)	20 (42.6)	5 (16.1)	19 (44.2)	**0.040*^a^***
β-lactam/β-lactamase Inhibitor	62 (40.5)	10 (31.3)	18 (38.3)	21 (67.7)	13 (30.2)	**0.005*^cg^***
Carbapenems	19 (12.4)	3 (9.4)	6 (12.8)	5 (16.1)	5 (11.6)	0.876
Quinolones	16 (10.5)	6 (18.8)	5 (10.6)	1 (3.2)	4 (9.7)	0.228
Aminoglycosides	7 (4.6)	1 (3.1)	3 (6.4)	2 (6.5)	1 (2.3)	0.726
Nitroimidazoles	11 (7.2)	0 (0)	7 (14.9)	2 (6.5)	2 (4.7)	**0.036*^a^***
Others	7 (4.6)	1 (3.1)	5 (10.6)	0 (0)	1 (2.3)	0.086
Single DAT, No. (%)						
Second/third-generation cephalosporins	46 (30.1%)	11 (34.4)	12 (25.5)	6 (19.4)	17 (39.5)	0.234
β-lactam/β-lactamase Inhibitor	48 (31.4)	8 (25.0)	12 (25.5)	17 (54.8)	11 (25.6)	**0.019*^a^***
Carbapenems	19 (12.4)	1 (3.1)	4 (8.5)	6 (19.4)	8 (18.6)	0.080
Quinolones	21 (13.7)	4 (12.5)	10 (21.3)	2 (6.5)	5 (11.6)	0.276
Aminoglycosides	8 (5.2)	3 (9.4)	2 (4.3)	2 (6.5)	1 (2.3)	0.571
Others	4 (2.6)	1 (3.1)	1 (2.1)	1 (3.2)	1 (2.3)	0.987
Combined DAT, No. (%)						
Combination of two antimicrobials	36 (23.5)	9 (28.1)	13 (27.7)	7 (22.6)	7 (16.3)	0.553
Combination of ≥ triple antimicrobials	4 (2.6)	0 (0)	3 (6.4)	1 (3.2)	0 (0)	0.117
Tumor treatment, No. (%)						
Radiotherapy	27 (17.6)	6 (18.8)	7 (14.9)	2 (6.5)	12 (27.9)	0.109
Chemotherapy	96 (62.7)	23 (71.9)	32 (68.1)	14 (45.1)	27 (62.8)	0.121
Immunotherapy	50 (32.7)	9 (28.1)	16 (34.0)	10 (32.3)	15 (34.9)	0.932
Targeted therapy	73 (47.7)	19 (59.4)	23 (48.9)	14 (45.2)	17 (39.5)	0.391
Herbal therapy	40 (26.1)	10 (31.3)	14 (29.8)	6 (19.4)	10 (23.3)	0.641
Concomitant drugs, No. (%)						
Immunosuppressant	121 (79.1)	23 (71.9)	37 (78.7)	22 (71.0)	39 (90.7)	0.124
Hormone	130 (85.0)	24 (75.0)	41 (87.2)	26 (83.9)	39 (90.7)	0.282
Clinical outcomes						
LOS in ICU, median (IQR)	0 (0, 0)	0 (0, 0)	0 (0, 0)	0 (0, 0)	0 (0, 0)	0.759
ICU, No. (%)	13 (8.5)	4 (12.5)	4 (8.5)	2 (6.5)	3 (7.0)	0.826
LOS, median (IQR)	12 (9, 23)	12 (7, 20)	12 (8, 22)	14 (10, 29)	12 (9, 29)	0.350
LOS after infection	9 (5, 16)	9 (5, 18)	8 (5, 13)	12 (8, 18)	9 (5, 15)	0.301
Clinical stability, No. (%)	122 (79.7)	29 (90.6)	35 (74.5)	21 (67.7)	37 (86.0)	0.073
On the mend, No. (%)	9 (5.9)	1 (3.1)	1 (2.1)	3 (9.7)	4 (9.7)	0.314
Deterioration, No. (%)	13 (8.5)	2 (6.3)	4 (8.5)	4 (12.9)	3 (7.0)	0.792
Automatic discharge or transfer, No. (%)	26 (17.0)	2 (6.3)	11 (23.4)	8 (25.8)	5 (11.6)	0.087
Readmission, No. (%)	97 (63.4)	26 (81.3)	30 (63.8)	14 (45.2)	27 (62.8)	**0.031*^c^***
Reinfection, No. (%)	27 (17.6)	7 (21.9)	4 (8.5)	8 (25.8)	8 (18.6)	0.209
Palliative care, No. (%)	11 (7.2)	2 (6.3)	3 (6.4)	2 (6.5)	4 (9.7)	0.944

*P values are obtained using the chi-square for categorical variables and Kruskal-Wallis test for continuous variables. ^#^1 dollar = 7.1138 Chinese Yuan as of September, 2025. Data are represented as No. (%), IQR and mean ± SD. Bold values indicate statistical significance (*P* < 0.05).

^a^No significant difference between any of the pairs in *post hoc* analysis.

^b^Significantly different between lung cancer and gastrointestinal cancer groups.

^c^Significantly different between lung cancer and hepatobiliary and pancreatic cancer groups.

^d^Significantly different between lung cancer and others groups.

^e^Significantly different between gastrointestinal cancer and hepatobiliary and pancreatic cancer groups.

^f^Significantly different between gastrointestinal cancer and others groups.

^g^Significantly different between hepatobiliary and pancreatic cancer and others groups.

Mon, monocytes; Cr, creatinine; Bun, blood urea nitrogen.

### 3.4 Treatment patterns and clinical outcomes

Compared with uninfected patients (**Table 1**), those with KP infection underwent chemotherapy less frequently but received radiotherapy more often (all *P* < 0.05). The concomitant use of immunosuppressant and hormone was also significantly higher in the KP group (all *P* < 0.001). KP infection was also associated with worse clinical outcomes, including longer hospital stays, higher rates of ICU admission, and poorer discharge status, with increased clinical deterioration and transfer rates and fewer stable discharges (all *P* < 0.05).

**Figure 3:**
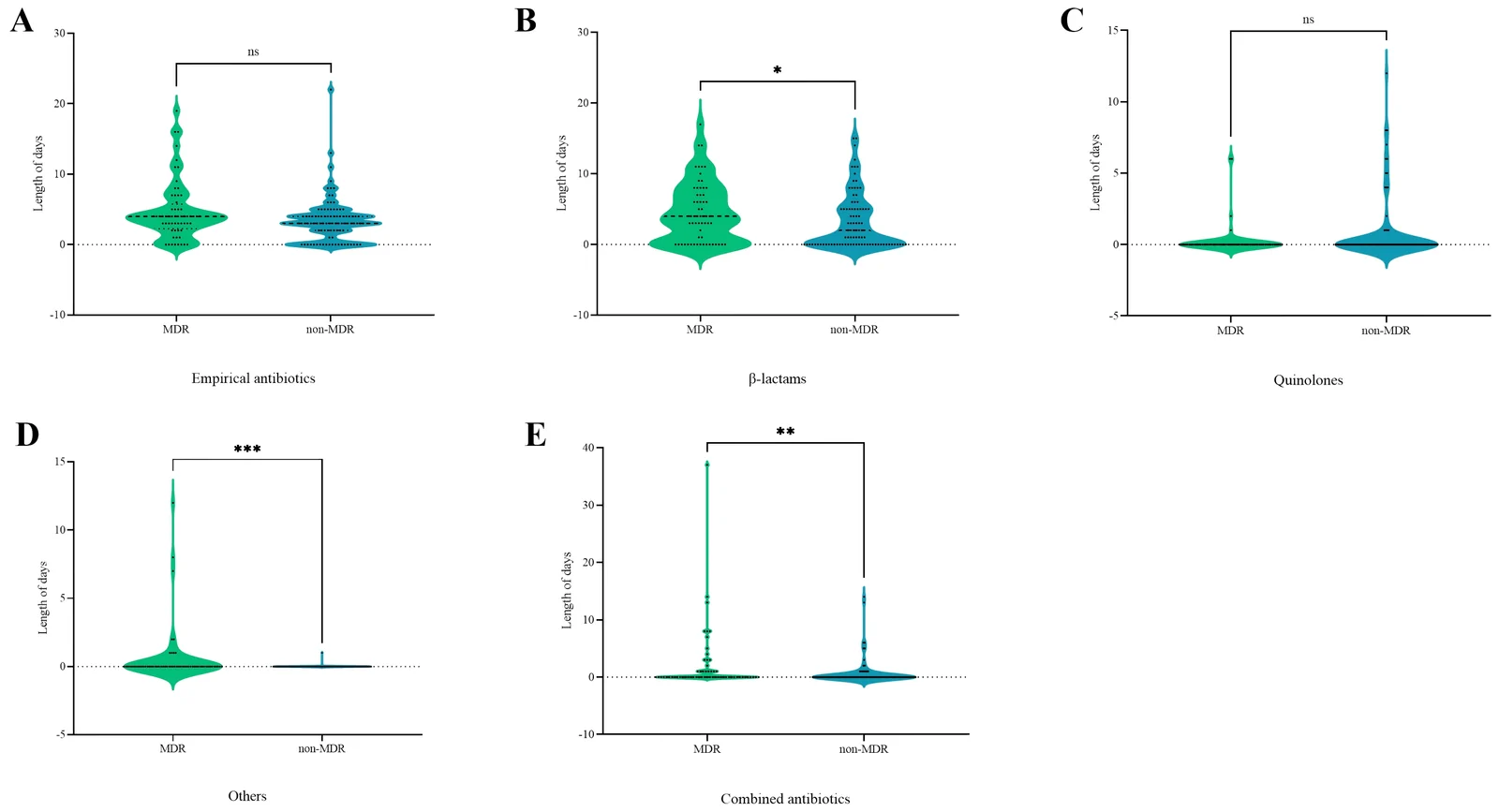
Violin plot of duration of antibiotic therapy in the MDR-KP and non-MDR-KP groups. **(A)** Empirical antibiotics, **(B)** β-lactams, **(C)** Quinolones, **(D)** Others and **(E)** Combined antibiotics for patient infection with MDR-KP. *P* values were obtained using the Mann–Whitney *U* test. Statistical significance between the two groups is denoted as follows: NS, not significant; *P < 0.05; **P < 0.01; and ***P < 0.001.

Among KP-positive cases, EAT was frequently administered, with no significant differences between resistance groups. However, β-lactam/β-lactamase inhibitor combinations were more frequently used in both MDR-KP and ESBL-KP groups (all *P* < 0.05). During definitive antibiotic therapy (DAT), aminoglycoside use was higher in both MDR-KP and ESBL-KP groups (all *P* < 0.05) (**Table 2**; **Supplementary Table S3**). Combination regimens were also more common in the MDR-KP group, including two-drug therapy (*P* = 0.022) and triple-or-more antimicrobial therapy (*P* = 0.045) (**Table 2**). Correspondingly, treatment duration for β-lactams and combination therapies was significantly longer in the MDR-KP group (all *P* < 0.05) (**Figure 3**). In the ESBL-KP group, although differences in combination therapy and treatment duration were not statistically significant, a trend toward more complex antimicrobial regimens was observed (**Supplementary Figure 1**). No significant differences were found between resistance groups in cancer treatment, concomitant medication use, or clinical outcomes (**Table 2**; **Supplementary Table 3**).

### 3.5 Independent predictors identified by multivariate analysis

**Figure 4:**
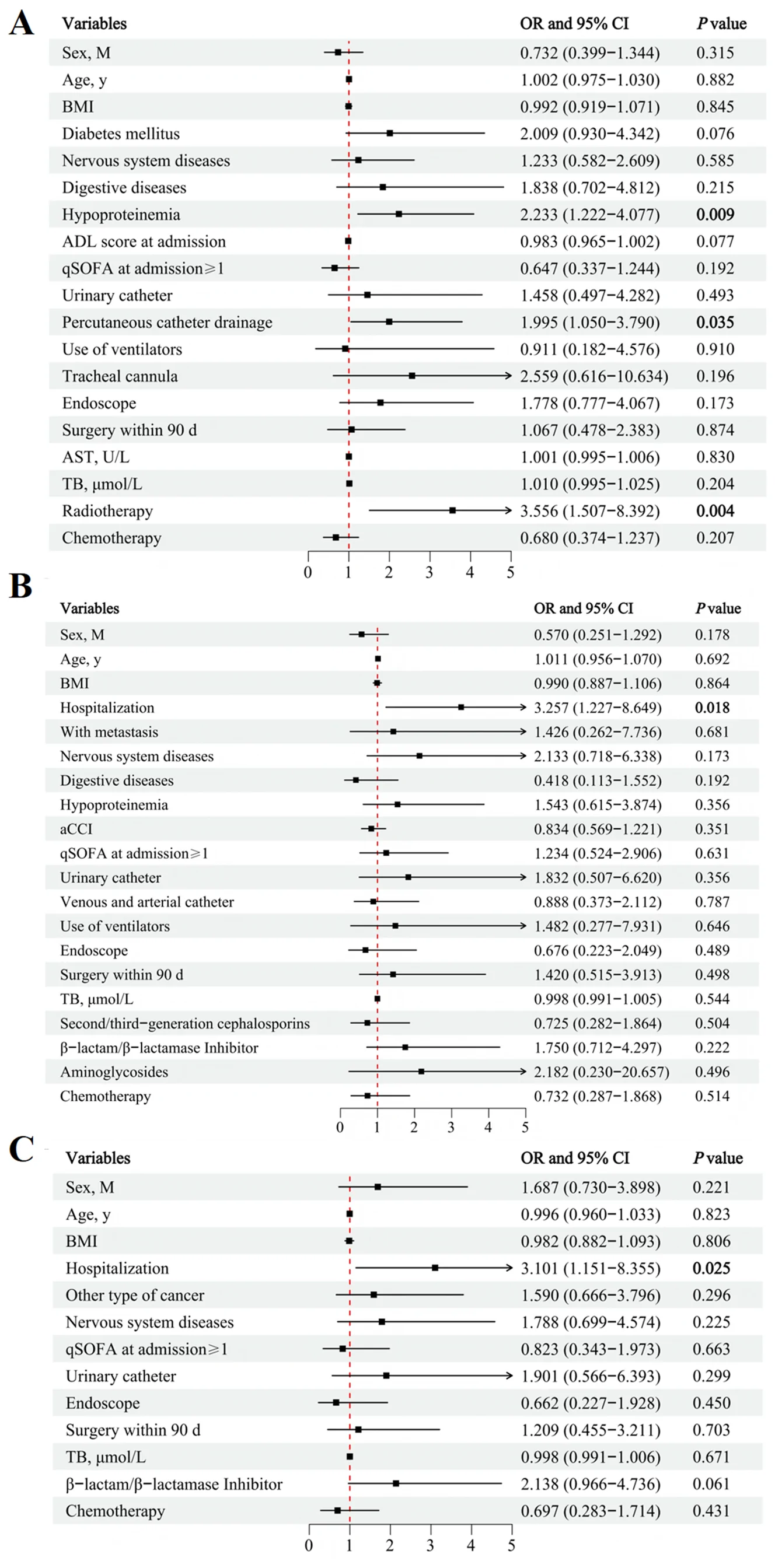
Multivariable logistic regression analysis of risk factors associated with KP infection. **(A)** Multivariate logistic regression analysis of the KP and uninfected groups. **(B)** Multivariate logistic regression analysis of the MDR-KP and non-MDR-KP groups. **(C)** Multivariate logistic regression analysis of the ESBL-KP and non-MDR-KP groups. Bold indicates *P* values <0.05 are statistically significant.

Multivariate logistic regression identified three factors independently associated with KP infection: hypoproteinemia (OR = 2.233, 95% CI 1.222-4.077, *P* = 0.009), percutaneous catheter drainage (OR = 1.995, 95% CI 1.050-3.790, *P* = 0.035), and radiotherapy (OR = 3.556, 95% CI 1.507-8.392, *P* = 0.004) (**Figure 4A**). In addition, recent hospitalization was identified as a significant factor contributing to both MDR-KP (OR = 3.257, 95% CI 1.227-8.649, *P* = 0.021) and ESBL-KP infections (OR = 3.101, 95% CI 1.151-8.355, *P* = 0.025) (**Figures 4B, C**).

### 3.6 Development and validation of a predictive nomogram

**Figure 5:**
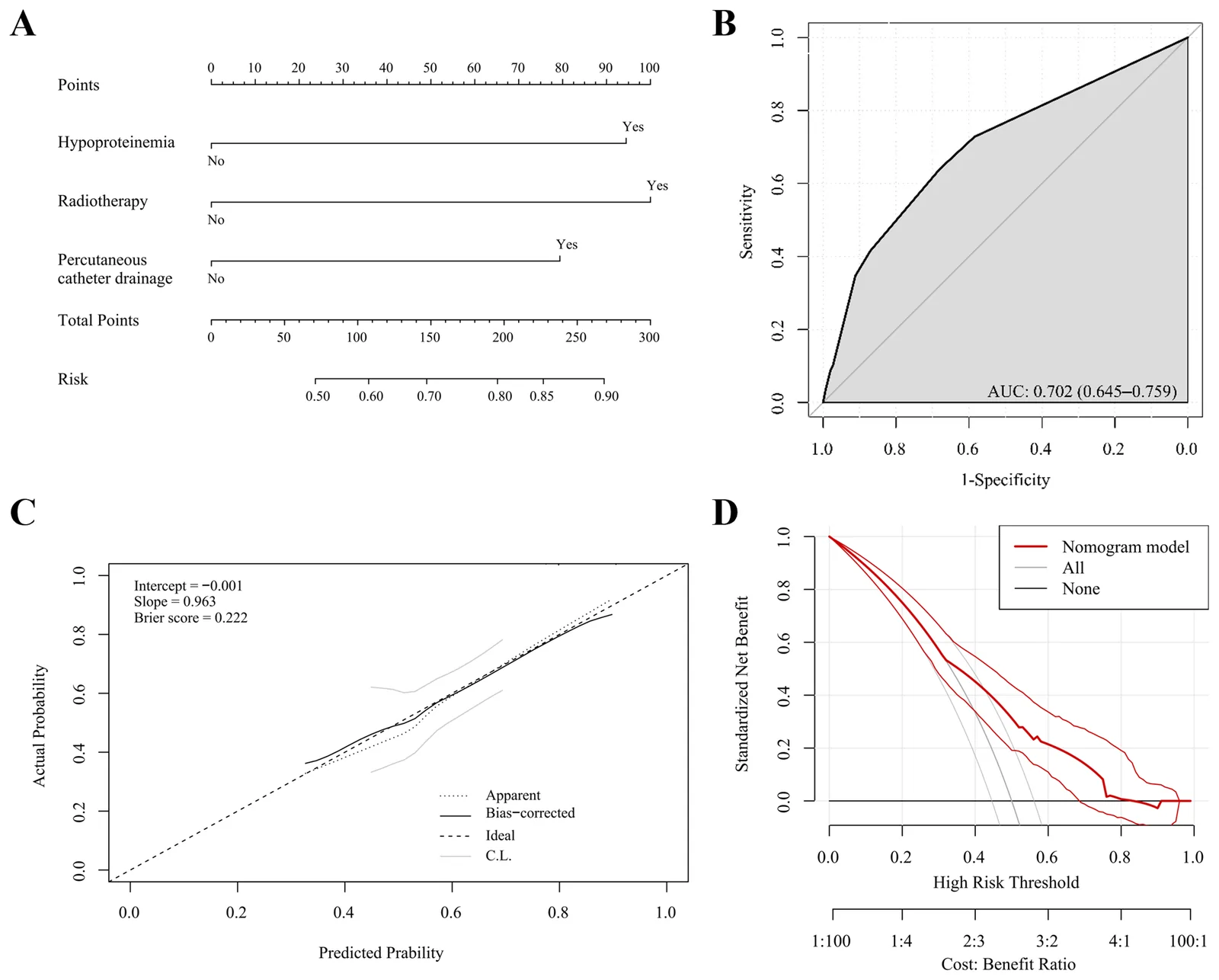
Risk prediction model based on logistic regression results and its effectiveness evaluation. **(A)** Nomogram based on hypoproteinemia, radiotherapy, and percutaneous catheter drainage. **(B)** Receiver-operating characteristic curve for predicting KP infection risk. **(C)** Bootstrap-corrected calibration plot with Brier score, calibration intercept, and slope. **(D)** Decision curve analysis for the nomogram predicting KP infection.

A nomogram was developed to estimate the likelihood of KP infection in cancer patients based on these identified clinical factors (**Figure 5A**). The model demonstrated acceptable discriminative ability, with an AUC of 0.702 (95% CI: 0.645-0.759) (**Figure 5B**). Calibration was acceptable, with a Brier score of 0.222, intercept of -0.001, and slope of 0.963 (**Figure 5C**). DCA further suggested the potential clinical utility of the nomogram, showing a higher net benefit than the “treat-all” or “treat-none” strategies over a wide range of threshold probabilities (**Figure 5D**).

## 4 Discussion

In this retrospective study, we comprehensively analyzed the clinical characteristics, antimicrobial resistance profiles, and risk factors associated with KP infection in cancer patients. Multivariate analysis identified hypoproteinemia, percutaneous catheter drainage, and radiotherapy as independent clinical factors correlated with KP infection. These findings collectively imply that impaired host immunity, frequent invasive procedures, and cumulative treatment exposures likely act synergistically to elevate the risk of infection and drive the emergence of antimicrobial resistance in oncological settings (Rolston, 2017).

Malnutrition is highly prevalent among cancer patients, occurring in 25-80% of individuals depending on tumor type, stage, and treatment (Robien and Wopat, 2025). Hypoalbuminemia, a common indicator of nutritional deficit and systemic inflammation, can compromise immune competence, tissue repair, and mucosal integrity, thereby potentially facilitating bacterial translocation and subsequent infection (Soeters et al., 2019). Percutaneous catheter drainage likewise identifies patients with disrupted anatomical barriers and complex underlying disease (Gudiol et al., 2016). Radiotherapy—but not chemotherapy—was independently associated with KP infection. Radiation-related tissue injury and mucosal-barrier disruption provide a plausible explanation (Wang et al., 2025b; Hauer-Jensen et al., 2014), although radiotherapy may also capture differences in tumor burden, tumor site, treatment intent, or treatment intensity. Regardless of mechanism, this association identifies a clinically recognizable subgroup that may warrant closer infection surveillance; it should not be interpreted as evidence that radiotherapy causes KP infection. Moreover, a considerable proportion of patients in our cohort had completed chemotherapy prior to hospitalization, which may explain why it did not show a significant independent association in the multivariate model (Wang et al., 2025b).

Recent hospitalization was associated with both MDR-KP and ESBL-KP, consistent with the role of repeated healthcare exposure in acquisition of resistant organisms (Çölkesen et al., 2023; Zhu et al., 2020). The nomogram uses three routinely available variables and may facilitate early risk stratification. Its discrimination was moderate, and its endpoint and population differ from models developed for ICU mortality, MDR coinfection, or pediatric CRKP (Song et al., 2025; Wang et al., 2024; Li et al. 2023a). Accordingly, the model may help prioritize monitoring and review of empirical therapy, but it is not intended to determine antimicrobial treatment on its own. External validation and additional predictors are needed before routine implementation.

We also observed a high proportion of respiratory tract infections and a predominance of MDR-KP and ESBL-KP among urinary isolates. The high frequency of respiratory KP infections may reflect impaired mucociliary clearance, prolonged immobilization, and frequent use of invasive respiratory support in hospitalized cancer patients (Hanania et al., 2019; Whitsett, 2018). The enrichment of MDR and ESBL strains in urinary specimens likely results from selective antibiotic pressure and the common use of indwelling catheters in this population, consistent with reports from China and Thailand (Song et al., 2025; Ponyon et al., 2022).

KP infections was most frequently among patients with gastrointestinal cancer. The gastrointestinal tract is a primary reservoir for KP, and cancer-related dysbiosis—driven by chemotherapy, radiotherapy, antibiotics, and malnutrition—can diminish colonization resistance, enabling pathogen overgrowth and translocation (Adelman et al., 2020). A healthy gut microbiome protects against infection by reinforcing epithelial barrier function, modulating immune responses, and competitively inhibiting pathogens (Sepich-Poore et al., 2021; Ducarmon et al., 2019). In contrast, dysbiosis weakens these protective mechanisms, allowing enteric bacteria such as KP to proliferate and disseminate to extraintestinal sites (Fernandes et al., 2022). This observation is biologically plausible but should be tested in prospective studies that measure colonization and microbiome changes directly.

MDR-KP infection was accompanied by higher inflammatory indices, more combination therapy, longer treatment, greater antibiotic costs (Wang et al., 2025c). The nine CRKP cases included XDR and PDR phenotypes, frequent ICU care and readmission, and two episodes of clinical deterioration; these observations are clinically informative but remain descriptive because of the small subgroup. In the analysis that excluded CRKP, ESBL-KP and non-MDR-KP groups had similar recorded clinical outcomes, although ESBL-KP remained associated with higher antimicrobial costs. These findings emphasize the management burden of resistance without implying that resistance phenotype alone determined outcome. At a broader level, antimicrobial resistance substantially increases clinical and economic burden worldwide (Antimicrobial Resistance Collaborators, 2022).

This study has several limitations. Its single-center retrospective design and the local nature of antimicrobial epidemiology limit generalizability, and the nomogram received internal but not external validation. Tumor stage, Eastern Cooperative Oncology Group performance status, treatment intensity, neutropenia duration, previous antimicrobial exposure, and prior colonization with resistant organisms were unavailable, leaving residual confounding. Molecular resistance-gene testing, multilocus sequence typing, and virulence profiling were not performed, precluding assessment of clonal transmission or mechanism. Unequal site-specific sample sizes reduced precision for some comparisons, and the absence of post-discharge follow-up prevented survival analysis. These limitations define priorities for multicenter prospective validation rather than negating the clinical patterns observed here.

## 5 Conclusion

This study provides a comprehensive clinical and microbiological characterization of KP infections in cancer patients, highlighting the relevant roles of host vulnerability, invasive procedures, and antimicrobial resistance. Hypoproteinemia, percutaneous catheter drainage, and radiotherapy emerged as potential independent risk factors associated with KP infection, whereas recent hospitalization was associated with MDR-KP and ESBL-KP phenotypes. Variability in resistance patterns across infection sites and cancer types supports the need for more tailored empirical therapy. The internally validated nomogram provides a practical basis for early risk stratification and should next be evaluated in external multicenter cohorts with additional clinical and molecular predictors.

## Data Availability

The raw data supporting the conclusions of this article will be made available by the authors, without undue reservation.
