# Supplementary material for: Risk factors, resistance profiles, and clinical outcomes of Klebsiella pneumoniae infection in cancer patients: a retrospective study
Source: Front Cell Infect Microbiol. 2026 Aug 26;16:1908965. doi: 10.3389/fcimb.2026.1908965 (PMC13553344; doi:10.3389/fcimb.2026.1908965)
Supplement: Supplementary file 1 [file Supplementaryfile1.docx]

**Table of Contents**

**Supplementary Table S1.** Sample size of antibiotic testing across infection sites and cancer types.

**Supplementary Table S2.** Baseline characteristics before and after propensity score matching.

**Supplementary Table S3.** Comparison of clinical characteristics between ESBL-KP and non-MDR-KP groups.

**Supplementary Table S4.** Clinical characteristics, antimicrobial resistance phenotypes, and outcomes of the nine patients with CRKP infection..

**Supplementary Figure S1.** Violin plot of duration of antibiotic therapy in the ESBL-KP and non-MDR-KP groups.

Supplementary Table S1. Sample size of antibiotic testing across infection sites and cancer types.

| **Antibiotics** | **Total (n=153)** | **Infection site** | | | | **Cancer type** | | | |
| --- | --- | --- | --- | --- | --- | --- | --- | --- | --- |
|  |  | Respiratory tract (n=93) | Unrinary tract (n=22) | Bloodstream (n=16) | Others (n=22) | Lung cancer (n=32) | Gastrointestinal cancer (n=47) | Hepatobiliary and Pancreatic cancer (31) | Others (n=43) |
| ETP | 138 | 89 | 19 | 12 | 18 | 29 | 44 | 26 | 39 |
| TGC | 127 | 78 | 17 | 13 | 19 | 30 | 35 | 28 | 34 |
| TMZ-SMZ | 151 | 93 | 22 | 16 | 20 | 32 | 47 | 29 | 43 |
| SCF | 137 | 85 | 19 | 13 | 20 | 29 | 40 | 29 | 39 |
| AMK | 149 | 92 | 21 | 16 | 20 | 31 | 47 | 29 | 42 |
| TZP | 150 | 92 | 22 | 16 | 20 | 31 | 47 | 29 | 43 |
| CAZ | 151 | 93 | 22 | 16 | 20 | 32 | 47 | 29 | 43 |
| FEP | 151 | 93 | 22 | 16 | 20 | 32 | 47 | 29 | 43 |
| IMI | 149 | 92 | 22 | 15 | 20 | 32 | 47 | 28 | 42 |
| LVX | 150 | 93 | 21 | 16 | 20 | 32 | 46 | 29 | 43 |
| FOX | 111 | 72 | 15 | 10 | 14 | 22 | 30 | 23 | 36 |
| CRO | 142 | 89 | 21 | 14 | 18 | 31 | 42 | 27 | 42 |
| CXM | 135 | 85 | 19 | 13 | 18 | 28 | 38 | 29 | 40 |
| ATM | 116 | 71 | 16 | 14 | 15 | 28 | 33 | 24 | 31 |
| TOB | 113 | 70 | 16 | 13 | 14 | 27 | 33 | 22 | 31 |
| CIP | 113 | 70 | 15 | 14 | 14 | 27 | 33 | 23 | 30 |
| MEM | 101 | 63 | 13 | 12 | 13 | 25 | 25 | 23 | 28 |
| CTT | 110 | 69 | 16 | 12 | 13 | 27 | 30 | 23 | 30 |
| GEN | 110 | 69 | 16 | 13 | 12 | 27 | 30 | 23 | 30 |
| CFZ | 99 | 63 | 13 | 13 | 10 | 26 | 25 | 21 | 27 |
| CMZ | 99 | 66 | 13 | 9 | 11 | 27 | 22 | 22 | 28 |
| CTX | 91 | 57 | 12 | 10 | 12 | 24 | 21 | 21 | 25 |
| CZA | 86 | 57 | 9 | 10 | 10 | 25 | 18 | 19 | 24 |
| SAM | 110 | 69 | 16 | 13 | 12 | 28 | 30 | 23 | 29 |
| MIN | 93 | 63 | 11 | 9 | 10 | 25 | 23 | 21 | 24 |
| PIP | 99 | 63 | 13 | 11 | 12 | 25 | 22 | 24 | 28 |

Supplementary Table S2. Baseline characteristics before and after propensity score matching

| **Variables** | **Matched** | |
| --- | --- | --- |
|  | ***P* value** | **SMD** |
| Sex, M | 0.302 | 0.117 |
| Age, y | 0.259 | 0.125 |
| BMI | 0.598 | 0.047 |
| qSOFA at admission ≥1 | 0.894 | 0 |
| Lung cancer | 0.775 | 0.034 |
| Gastrointestinal cancer | 0.533 | 0.073 |
| Hepatobiliary and Pancreatic cancer | 0.884 | 0.017 |
| Others | 0.424 | 0.093 |
| With metastasis | 1.000 | 0 |

*P* values are obtained using chi-square test or fisher’s exact test for categorical variables and the independent-samples t-test or Mann-Whitney *U* for continuous variables.

Supplementary Table S3 Comparison of clinical characteristics between ESBL-KP and non-MDR-KP groups.

| **Variables** | **Total (n=144)** | **ESBL-KP (n=51)** | **non-MDR-KP (n=93)** | ***P* value*** |
| --- | --- | --- | --- | --- |
| Sex, M**, No. (%)** | 99 (68.8) | 30 (58.8) | 69 (74.2) | 0.057 |
| **Age, y, median (IQR)** | 68 (58, 73) | 68 (58, 73) | 68 (58, 74) | 0.584 |
| **BMI, median (IQR)** | 20.76 (18.37, 24.22) | 22.03 (19.37, 24.54) | 20.76 (18.08, 23.95) | 0.453 |
| **History, No. (%)** | | | | |
| Drinking | 39 (27.1) | 13 (25.5) | 26 (28.0) | 0.750 |
| Smoking | 46 (31.9) | 15 (19.4) | 31 (33.3) | 0.629 |
| Hospitalization | 98 (68.1) | 41 (80.4) | 57 (61.3) | **0.019** |
| Infection | 48 (33.3) | 18 (35.3) | 30 (32.3) | 0.712 |
| **Infection type, No. (%)** | | | | |
| Community acquired | 51 (35.4) | 17 (33.3) | 34 (36.6) | 0.699 |
| Healthcare associated | 65 (45.1) | 25 (49.0) | 40 (43.0) | 0.488 |
| Hospital acquired | 28 (19.4) | 9 (17.6) | 19 (20.4) | 0.687 |
| **Cancer type, No. (%)** | | | | |
| Lung cancer | 30 (20.8) | 10 (19.6) | 20 (21.5) | 0.789 |
| Gastrointestinal cancer | 46 (31.9) | 14 (27.5) | 32 (34.4) | 0.392 |
| Hepatobiliary and Pancreatic cancer | 27 (18.8) | 9 (17.6) | 18 (19.4) | 0.802 |
| Others | 42 (29.2) | 19 (37.3) | 23 (24.7) | 0.114 |
| With metastasis | 85 (59.0) | 27 (52.9) | 58 (62.4) | 0.271 |
| Comorbid conditions**, No. (%)** | | | | |
| Hypertension | 54 (37.5) | 21 (41.2) | 33 (35.5) | 0.500 |
| Diabetes mellitus | 31 (21.5) | 13 (25.5) | 18 (19.4) | 0.392 |
| Nervous system diseases | 31 (21.5) | 15 (19.4) | 16 (17.2) | 0.088 |
| Respiratory diseases | 29 (20.1) | 11 (21.6) | 18 (19.4) | 0.751 |
| Cardiovascular diseases | 28 (19.4) | 10 (19.6) | 18 (19.4) | 0.971 |
| Digestive diseases | 21 (14.6) | 5 (9.8) | 16 (17.2) | 0.229 |
| Chronic kidney diseases | 19 (13.2) | 9 (17.6) | 10 (10.8) | 0.242 |
| Chronic hepatic diseases | 31 (21.5) | 9 (17.6) | 22 (23.7) | 0.401 |
| **Hypoproteinemia, No. (%)** | 79 (54.9) | 30 (58.8) | 49 (52.7) | 0.479 |
| **aCCI, median (IQR)** | 8 (6, 9) | 8 (6, 9) | 8 (6, 9) | 0.332 |
| **ADL score at admission, median (IQR)** | 95 (80, 100) | 95 (70, 100) | 95 (80, 100) | 0.733 |
| **ADL score at discharge, median (IQR)** | 95 (75, 100) | 90 (65, 100) | 95 (80, 100) | 0.450 |
| **qSOFA at admission ≥1, No. (%)** | 40 (27.8) | 17 (33.3) | 23 (24.7) | 0.270 |
| **Invasive procedure, No. (%)** | | | | |
| Urinary catheter | 27 (18.8) | 14 (27.5) | 13 (14.0) | **0.048** |
| Percutaneous catheter drainage | 55 (38.2) | 19 (37.3) | 36 (38.7) | 0.864 |
| Venous and arterial catheter | 47 (32.6) | 18 (35.3) | 29 (31.2) | 0.615 |
| Use of ventilators | 15 (10.4) | 7 (13.7) | 8 (8.6) | 0.336 |
| Tracheal cannula | 15 (10.4) | 4 (7.8) | 11 (11.8) | 0.454 |
| Endoscope | 29 (20.1) | 7 (13.7) | 22 (23.7) | 0.155 |
| Others | 8 (5.6) | 4 (7.8) | 4 (4.3) | 0.612 |
| Invasive procedure within 7 d | 49 (34.0) | 18 (35.3) | 31 (33.3) | 0.812 |
| Surgery within 90 d | 43 (29.9) | 20 (39.2) | 23 (24.7) | 0.069 |
| **Laboratory findings at admission, median (IQR)** | | | | |
| WBC, ×10^9/L | 6.46 (4.27, 9.76) | 8.01 (4.66, 10.05) | 5.96 (4.20, 9.32) | 0.064 |
| Neu, ×10^9/L | 4.46 (2.80, 7.99) | 5.65 (3.29, 9.07) | 4.16 (2.76, 7.16) | 0.068 |
| Lym, ×10^9/L | 0.80 (0.46, 1.39) | 0.85 (0.45, 1.40) | 0.77 (0.46, 1.36) | 0.515 |
| Mon, ×10^9/L | 0.39 (0.22, 0.57) | 0.42 (0.27, 0.59) | 0.33 (0.20, 0.56) | 0.131 |
| PLT, ×10^9/L | 155 (113, 228) | 184 (129, 251) | 143 (98, 207) | **0.028** |
| Hb, g/L, (mean ± SD) | 107 ±20 | 105 ±19 | 108 ±21 | 0.386 |
| SII | 900.83 (442.32, 1856.81) | 1002.48 (515.87, 2171.35) | 768.45 (378.76, 1777.93) | 0.053 |
| NLR | 6.28 (2.89, 10.94) | 7.75 (3.80, 11.77) | 6.15 (2.57, 10.74) | 0.279 |
| PLR | 200.47 (123.70, 296.35) | 213.73 (136.37, 338.10) | 188.33 (119.76, 284.45) | 0.294 |
| MLR | 0.47 (0.26, 0.74) | 0.50 (0.33, 0.91) | 0.46 (0.24, 0.68) | 0.163 |
| ALT, U/L | 18.5 (11.7, 30.6) | 19.2 (11.6, 34.0) | 18.0 (11.7, 30.2) | 0.773 |
| AST, U/L | 26.5 (18.0, 39.8) | 26.5 (17.5, 38.1) | 26.4 (18.1, 45.3) | 0.897 |
| TB, μmol/L | 13.98 (9.72, 20.63) | 11.05 (8.05, 20.46) | 14.74 (10.47, 21.50) | **0.029** |
| TP, g/L | 61.0 (55.7, 65.7) | 62.2 (57.2, 66.8) | 59.9 (55.3, 65.5) | 0.256 |
| ALB, g/L | 34.9 (31.5, 38.6) | 34.7 (31.6, 37.5) | 35.2 (31.4, 39.7) | 0.368 |
| Cr, μmol/L | 62.5 (50.9, 77.8) | 63.5 (51.2, 83.4) | 60.0 (50.7, 77.8) | 0.511 |
| Bun, mmol/L | 5.15 (4.18, 7.52) | 5.56 (3.93, 7.94) | 5.05 (4.28, 7.08) | 0.552 |
| **Fever, No. (%)** | 56 (38.9) | 19 (37.3) | 37 (39.8) | 0.766 |
| Fever ≥ 72h | 31 (21.5) | 12 (23.5) | 19 (20.4) | 0.665 |
| **Bacteremia, No. (%)** | 14 (9.7) | 3 (5.9) | 11 (11.8) | 0.391 |
| **Shock, No. (%)** | 7 (4.9) | 2 (3.9) | 5 (5.4) | 1.000 |
| Related cost (dollars)**^#^, median (IQR)** | | | | |
| Total cost | 2576.6 (1559.9, 6192.5) | 2754.0 (1600.4, 6217.1) | 2560.4 (1524.3, 6298.4) | 0.952 |
| Medical fee | 1132.6 (555.6, 1873.7) | 1202.3 (545.6, 2347.2) | 1109.9 (580.4, 1801.1) | 0.836 |
| Antibacterial agent cost | 39.0 (7.8, 189.4) | 70.8 (25.2, 237.1) | 20.4 (2.9, 138.7) | **0.007** |
| **Treatment with antibiotics, No. (%)** | | | | |
| Empirical antibiotic therapy | 117 (81.3) | 44 (86.3) | 73 (78.5) | 0.253 |
| Second/third-generation cephalosporins | 53 (36.8) | 17 (33.3) | 32 (38.7) | 0.522 |
| β-lactam/β-lactamase inhibitor | 57 (39.6) | 27 (52.9) | 30 (32.3) | **0.015** |
| Carbapenems | 12 (8.3) | 3 (5.9) | 9 (9.7) | 0.636 |
| Quinolones | 16 (11.1) | 5 (9.8) | 11 (11.8) | 0.712 |
| Aminoglycosides | 3 (2.1) | 1 (2.0) | 2 (2.2) | 1.000 |
| Nitroimidazoles | 11 (7.6) | 5 (9.8) | 6 (6.5) | 0.692 |
| Others | 6 (4.2) | 2 (3.9) | 4 (4.3) | 1.000 |
| **Single DAT, No. (%)** | | | | |
| Second/third-generation cephalosporins | 46 (31.9) | 17 (33.3) | 29 (31.2) | 0.791 |
| β-lactam/β-lactamase inhibitor | 44 (30.6) | 17 (33.3) | 27 (29.0) | 0.592 |
| Carbapenems | 15 (10.4) | 5 (9.8) | 10 (10.8) | 0.859 |
| Quinolones | 21 (14.6) | 5 (9.8) | 16 (17.2) | 0.229 |
| Aminoglycosides | 6 (4.2) | 5 (9.8) | 1 (1.1) | **0.038** |
| Others | 3 (2.1) | 2 (3.9) | 1 (1.1) | 0.594 |
| **Combined DAT, No. (%)** | | | | |
| Combination of two antimicrobials | 32 (22.2) | 16 (31.4) | 16 (17.2) | 0.050 |
| Combination of ≥ triple antimicrobials | 3 (2.1) | 3 (5.9) | 0 (0) | 0.079 |
| **Tumor treatment, No. (%)** | | | | |
| Radiotherapy | 25 (17.4) | 8 (15.7) | 17 (18.3) | 0.694 |
| Chemotherapy | 94 (65.3) | 31 (60.8) | 63 (67.7) | 0.402 |
| Immunotherapy | 49 (34.0) | 21 (41.2) | 28 (30.1) | 0.180 |
| Targeted therapy | 72 (50.0) | 28 (54.9) | 44 (47.3) | 0.384 |
| Herbal therapy | 40 (27.8) | 15 (27.8) | 25 (26.9) | 0.746 |
| **Concomitant drugs, No. (%)** | | | | |
| Immunosuppressant | 112 (77.8) | 43 (84.3) | 69 (74.2) | 0.162 |
| Hormone | 121 (84.0) | 46 (90.2) | 75 (80.6) | 0.135 |
| **Clinical outcomes** | | | | |
| LOS in ICU, median (IQR) | 0 (0, 0) | 0 (0, 0) | 0 (0, 0) | 0.540 |
| ICU, No. (%) | 9 (6.3) | 4 (7.8%) | 5 (5.4) | 0.822 |
| LOS, median (IQR) | 12 (8, 21) | 13 (8, 29) | 12 (8, 20) | 0.494 |
| LOS after infection, median (IQR) | 9 (5, 14) | 10 (6, 14) | 9 (5, 16) | 0.581 |
| Clinical stability, No. (%) | 116 (80.6) | 40 (78.4) | 76 (81.7) | 0.633 |
| On the mend, No. (%) | 7 (4.9) | 2 (3.9) | 5 (5.4) | 1.000 |
| Deterioration (%) | 11 (7.6) | 4 (7.8) | 7 (7.5) | 1.000 |
| Automatic discharge or transfer, No. (%) | 24 (16.7) | 10 (19.6) | 14 (15.1) | 0.483 |
| Readmission, No. (%) | 91 (63.2) | 31 (60.8) | 60 (64.5) | 0.657 |
| Reinfection, No. (%) | 25 (17.4) | 8 (15.7) | 17 (18.3) | 0.694 |
| Palliative care, No. (%) | 11 (7.6) | 3 (5.9) | 8 (8.6) | 0.795 |

* *P* values are obtained using chi-square test or fisher’s exact test for categorical variables and the independent-samples t-test or Mann-Whitney *U* for continuous variables ^#^ 1 dollar = 7.1138 Chinese Yuan as of September, 2025. Data are represented as No. (%), IQR and mean ± SD. Bold values indicate statistical significance (*P* < 0.05).

Abbreviations: Mon, monocytes; Cr, creatinine; Bun, blood urea nitrogen.

Supplementary Table S4. Clinical characteristics, antimicrobial resistance phenotypes, and outcomes of the nine patients with CRKP infection.

| **Patient** | **Sex** | **Age** | **Cancer type** | **ICU** | **Infection site** | **Main Comorbidities** | **Outcome** | **Resistance phenotype** |
| --- | --- | --- | --- | --- | --- | --- | --- | --- |
| 1 | Female | 78 | Hepatobiliary and Pancreatic cancer | No | Intra-abdominal infection | Chronic hepatic diseases | Deterioration; Automatic discharge or transfer | CRKP; PDR |
| 2 | Male | 71 | Gastrointestinal cancer | Yes | Intra-abdominal infection | Hypertension; Respiratory diseases; Cardiovascular diseases | Clinical stability; Readmission | CRKP; XDR |
| 3 | Female | 76 | Hepatobiliary and Pancreatic cancer | No | Bloodstream | Respiratory diseases; Chronic hepatic diseases | On the mend | CRKP; MDR |
| 4 | Male | 44 | Hepatobiliary and Pancreatic cancer | Yes | Bloodstream | Chronic hepatic diseases | Reinfection; Readmission; Clinical stability | CRKP; XDR |
| 5 | Male | 77 | Lung cancer | Yes | Respiratory tract | Diabetes mellitus; Respiratory diseases; Cardiovascular diseases; Chronic kidney diseases | Readmission; Deterioration; Automatic discharge or transfer | CRKP; MDR |
| 6 | Male | 57 | Hepatobiliary and Pancreatic cancer | No | Respiratory tract | Chronic hepatic diseases | Clinical stability; Readmission | CRKP; MDR |
| 7 | Male | 74 | Gastrointestinal cancer | No | Urinary tract | Hypertension; Respiratory diseases; Chronic hepatic diseases; Nervous system diseases | Clinical stability; Readmission | CRKP; XDR |
| 8 | Male | 54 | Lung cancer | Yes | Respiratory tract | Respiratory diseases; Nervous system diseases | Reinfection; Clinical stability; Readmission | CRKP; MDR |
| 9 | Male | 70 | Prostate cancer | No | Urinary tract | Hypertension; Cardiovascular diseases; Nervous system diseases | Clinical stability | CRKP; MDR |

**Supplementary Figure S1. Violin plot of duration of antibiotic therapy in the ESBL-KP and non-MDR-KP groups. (A)** Empirical antibiotics, **(B)** β-lactams, **(C)** Quinolones, **(D)** Others and **(E)** Combined antibiotics for patient infection with MDR-KP. Notes: *P* values were obtained using the Mann-Whitney *U* test. “NS” indicated no statistical difference between the two groups.
